# Gallic acid attenuates diabetic cardiomyopathy by inhibiting ferroptosis and protecting mitochondria via the TSPO/FTMT pathway

**DOI:** 10.3389/fphar.2025.1661144

**Published:** 2025-12-03

**Authors:** Chenchao Zou, Huaihan Xu, Fajia Hu, Yuxian Yu, Lanxiang Liu, Xiuqi Wang, Zheyu Zhang, Huaxi Zou, Jichun Liu, Huang Huang, Songqing Lai

**Affiliations:** 1 Department of Cardiovascular Surgery, The Second Affiliated Hospital, Jiangxi Medical College, Nanchang University, Nanchang, China; 2 Department of Cardiovascular Surgery, The First Affiliated Hospital, Jiangxi Medical College, Nanchang University, Nanchang, China; 3 Medical Innovation Experimental Program, Huan Kui College, Nanchang University, Nanchang, China

**Keywords:** phytochemical, diabetic cardiomyopathy, mitochondrial homeostasis, ferroptosis, reactive oxygen species

## Abstract

**Purpose:**

Diabetic cardiomyopathy (DCM), which is diabetes mellitus-induced cardiomyopathy, significantly elevates the risk of heart failure and sudden cardiac death. No specific treatments for DCM are currently available. Gallic acid (GA) is a polyhydroxyphenolic compound that has been shown to inhibit ferroptosis and maintain mitochondrial homeostasis, with potential therapeutic effects in various cardiac diseases. However, its specific role and underlying mechanisms in DCM remain unexplored.

**Methods:**

An *in vitro* model was established using H9C2 cells pretreated with high glucose plus palmitate, and an *in vivo* type 2 diabetes mellitus model generated by treating rats with streptozotocin-induced and feeding a high-fat diet. The protective effects of GA and its mechanism of action were evaluated using various methods, including flow cytometry, Western blotting (WB), and transmission electron microscopy. Bioinformatics analysis identified potential target genes for GA’s cardioprotection, which were subsequently validated using pAD/TSPO (for overexpression) and pAD/FTMT-shRNA (for silencing) constructs.

**Results:**

GA treatment decreased PTGS2, lactate dehydrogenase, malondialdehyde, ferrous iron, ROS, and oxidized glutathione disulfide (GSSG) levels and increased cell viability, glutathione (GSH) levels, the GSH/GSSG ratio, and GPX4 protein levels in the injury models. GA markedly attenuated mitochondrial ultrastructural damage and promoted mitochondrial homeostasis. These protective effects were abrogated by TSPO overexpression and FTMT silencing.

**Conclusion:**

GA was shown to attenuate diabetic cardiomyopathy by inhibiting ferroptosis and protecting mitochondria via the TSPO/FTMT signaling pathway.

## 1 Introduction

The global prevalence of diabetes mellitus is rapidly increasing, with projections indicating that it will affect 10.9% of the world’s population by 2045 (Saeedi et al., 2019). Furthermore, over one-third of patients with diabetes are estimated to develop diabetic cardiomyopathy (DCM) (Tong et al., 2019). DCM is defined as a structural and functional impairment of the myocardium caused by diabetes, independent of coronary artery disease, hypertension, and other structural heart diseases (Tan et al., 2020). The characteristic features of DCM include cardiac hypertrophy, myocardial fibrosis, and impaired cellular signaling (Yuan et al., 2023). DCM can significantly increase the risk of heart failure and sudden cardiac death in patients with diabetes (Lala et al., 2024). However, the precise pathogenesis of DCM remains incompletely understood, and specific therapeutic interventions for this prevalent cardiac complication are lacking.

Gallic acid (GA) is a naturally occurring polyhydroxyphenolic compound widely found in edible plants (Xiang et al., 2024). Studies have demonstrated that GA exerts effects such as anti-inflammatory (Bai J. et al., 2021) and antioxidant (Yang et al., 2023) effects and also inhibits ferroptosis (Hu et al., 2022) and maintains mitochondrial homeostasis (Kosuru et al., 2018), indicating its therapeutic potential for a range of heart diseases (Xiang et al., 2024; Yan et al., 2019; Das et al., 2024). Previous experiments in rats showed that GA could ameliorate endothelial dysfunction and hypotension in diabetes mellitus by upregulating plasma miR-24 and miR-126 levels (Ramezani Ali Akbari et al., 2019). Additionally, GA has been reported to attenuate cardiac hypertrophy and left ventricular dysfunction following cardiac ischemia-reperfusion injury in diabetic rats (Ramezani-Aliakbari et al., 2017). Despite these promising findings, research specifically investigating GA for treating DCM is limited, and its therapeutic potential in this context requires further investigation.

Ferroptosis is an iron-dependent form of cell death (Jiang et al., 2021), which is mainly characterized by the excessive accumulation of iron and lipid peroxides. It has been implicated in the pathogenesis of numerous cardiac diseases (Fang et al., 2023). Accumulating evidence indicates that ferroptosis contributes significantly to the development of DCM (Ke et al., 2023; Yan et al., 2023) and is closely associated with mitochondrial dysfunction (Glover et al., 2024). Distinct from other programmed cell deaths, mitochondria exhibit unique ultrastructural changes in ferroptosis, including mitochondrial atrophy, increased membrane density, and reduced cristae (Li et al., 2023). As the primary organelles responsible for cellular energy production through oxidative phosphorylation, mitochondria are critical for sustaining contractile function and viability in energy-demanding cardiomyocytes (Da Dalt et al., 2023). Notably, DCM is frequently characterized by impaired mitochondrial structure and function within cardiomyocytes. Thus, inhibiting ferroptosis and preserving mitochondrial homeostasis represent promising therapeutic targets for DCM.

Given the central role of ferroptosis and mitochondrial dysfunction in DCM, identifying key molecular targets that regulate these processes is crucial for developing effective therapies. In this context, two mitochondrial proteins have emerged as potential regulators: the translocator protein (TSPO) and mitochondrial ferritin (FTMT). Translocator protein (TSPO) is a membrane protein located in the mitochondrial membrane, consisting of 169 amino acids and five transmembrane α-helical structural domains (Baglini et al., 2024). In most tissues, it is predominantly expressed in mitochondria, more specifically at the contact site between the outer and inner mitochondrial membranes (Baglini et al., 2024). TSPO is involved in the regulation of a variety of cellular functions, such as apoptosis (Shoshan-Barmatz et al., 2019), ferroptosis (Zhang D. et al., 2023), cellular respiration and oxidative processes (Baglini et al., 2024), protein import, and ion transport (Baglini et al., 2024). Studies have shown that TSPO has diverse cardiac effects and is involved in a variety of cardiac diseases (Baglini et al., 2024; Bai X. et al., 2021; Bréhat et al., 2024). Mitochondrial ferritin (FTMT) is an important mitochondrial iron storage protein that is mainly expressed in highly metabolically active tissues such as the heart and brain (Yanatori et al., 2023). FTMT catalyzes the oxidation of ferrous iron to trivalent iron and stores iron in a soluble, non-toxic form (Yanatori et al., 2023). It has been reported to inhibit ferroptosis and maintain mitochondrial homeostasis (Yanatori et al., 2023; Zhang et al., 2022).

## 2 Materials and methods

### 2.1 Reagents and materials

Gallic acid (GA) was purchased from MedChemExpress. Ferroptosis inhibitor (Fer-1, GC10380) and ferroptosis inducer (erastin, GC16630) were purchased from Glpbio. Z-VAD-FMK (apoptosis inhibitor, HY-16658B) were obtained from MCE. Adenovirus pAD/FTMT-shRNA was purchased from Shanghai Genechem, and adenovirus pAD/TSPO was purchased from WZ Biosciences. Antibodies against GPX4 (R381958), FTL (R30039), and FTH (R23306) were purchased from Zen-Bioscience, antibodies against FTMT (MBS2002952) were purchased from MyBioSource, antibodies against TSPO (SA90-03) were purchased from HUABIO, and antibodies against PTGS2 (12375-1-AP) and β-actin (81115-1-RR) were bought from Proteintech Group. Goat anti-mouse (A0216) and goat anti-rabbit (A0208) secondary antibodies were purchased from Beyotime Biotechnology.

### 2.2 Animals and cells

Sprague-Dawley male rats were obtained from the animal center of Nanchang University (Nanchang, Jiangxi, China). The animal study procedures adhered to the guidelines set by the United States National Institutes of Health and were approved by the Animal Experimentation Ethics Committee of the First Affiliated Hospital of Nanchang University (CDYFY-IACUC-202502GR069). The rats were housed under controlled conditions, with a 12-h light-darkness cycle and were provided ample food and water.

H9C2 cells, derived from embryonic BDIX rat heart tissue, were obtained from the cell bank/stem cell bank in Beijing, China. The cells were cultured in Dulbecco’s modified Eagle medium (DMEM) containing 5.5 mM glucose, 10% fetal bovine serum (FBS; Gibco, Thermo Fisher Scientific) at 37 °C and 5% CO_2_, 21% O_2_, and 95% humidity.

### 2.3 *In vivo* experiments

#### 2.3.1 Experimental design

The rats were randomly assigned to five experimental groups: 1) control, 2) DCM, 3) DCM +25 mg/kg GA, 4) DCM +50 mg/kg GA, and 5) DCM +100 mg/kg GA. Type 2 diabetes mellitus (T2DM) was induced according to established methods (Wu et al., 2022). Following a 1-week acclimatization period, the rats received a high-fat/high-glucose diet for 4 weeks, followed by intraperitoneal injections of streptozotocin (STZ, 35 mg/kg/day in citrate buffer, pH 4.5). T2DM was confirmed when fasting blood glucose levels exceeded 16.7 mM for 3 consecutive days. Eight weeks post-T2DM confirmation, based on preliminary studies (Chen et al., 2009; Bouzghaya et al., 2020), the treatment groups received daily oral gavage doses of low (25 mg/kg), medium (50 mg/kg), or high (100 mg/kg) GA for 4 weeks.

#### 2.3.2 Echocardiography

Cardiac function was assessed under 1.5% isoflurane anesthesia using transthoracic echocardiography (Vevo2100 Imaging System, Visual Sonics, Inc.). Two-dimensional parasternal short-axis views were obtained to calculate the left ventricular ejection fraction (LVEF) and fractional shortening (LVFS).

#### 2.3.3 Biochemical measurements

Terminal blood samples were collected via cardiac puncture from anesthetized rats in each experimental group. Serum was isolated by centrifugation (3,000 × g, 15 min, 4 °C). Serum levels of lactate dehydrogenase (LDH), ferrous iron (Fe^2+^), creatine kinase-MB (CK-MB), and malondialdehyde (MDA) were measured using the D-lactate dehydrogenase assay kit (Beyotime, P0392S), ferric and ferrous ion assay kit (Beyotime, S1066S), ElaBoX^TM^Rat CK-MB1 ELISA Kit (Solarbio, SEKR-0059), and the lipid peroxidation MDA assay kit (Beyotime, S0131S) according to the manufacturers’ instructions.

#### 2.3.4 Hematoxylin and eosin, masson, and dihydroethidium assays

Following euthanasia via CO_2_ inhalation, hearts were excised to calculate the heart-to-body weight ratio. Tissues underwent fixation in 4% paraformaldehyde, paraffin-embedding, and sectioning. Consecutive sections were stained with hematoxylin and eosin (H&E), Masson stain and dihydroethidium (DHE). The stained sections were imaged under light microscopy (Olympus).

### 2.4 *In vitro* experiments

#### 2.4.1 Experimental design

H9C2 cells were randomly divided into nine experimental groups: 1) control: H9C2 cells cultured in a standard conditions, 2) high glucose plus palmitate (HG + PA): H9C2 cells exposed to 30 mM glucose and 300 μM palmitate for 24 h, The concentrations were determined based on previous studies (Zhang N. et al., 2023; Hsu et al., 2023; Zhang L. et al., 2023) and our preliminary experimental results (Supplementary Figure S1), 3) GA: H9C2 cells cultured with 1, 5, 10, 20, 40, or 80 μM GA for 24 h, 4) HG + PA + GA: H9C2 cells cultured with 1, 5, 10, 20, 40, or 80 μM GA and high glucose plus palmitate for 24 h, 5) HG + PA + Fer-1: H9C2 cells cultured with 1 μM Fer-1 and high glucose plus palmitate for 24 h, 6) erastin: H9C2 cells treated with 10 μM erastin for 24 h, 7) erastin + GA: H9C2 cells treated with 10 μM GA and 10 μM erastin for 24 h, 8) HG + PA + GA + pAD/TSPO: H9C2 cells transfected with pAD/TSPO before treating with 10 μM GA and high glucose plus palmitate for 24 h, and 9) HG + PA + GA + pAD/FTMT-shRNA: H9C2 cells transfected with pAD/FTMT-shRNA before treating with 10 μM GA and high glucose plus palmitate for 24 h.

#### 2.4.2 Cell viability and cytotoxicity

Cell viability was assayed using the Cell Counting Kit-8 (GlpBio, GK10001), and cytotoxicity was assayed using the LDH assay kit (Beyotime, C0016) according to the manufacturer’s instructions (Hu et al., 2023).

#### 2.4.3 Measurement of MDA and reduced glutathione/oxidized glutathione disulfide levels

MDA and reduced glutathione (GSH)/oxidized glutathione disulfide (GSSG) content in H9C2 cells was measured using a lipid peroxidation MDA assay kit (Beyotime, S0131S) and a GSH and GSSG assay kit (Beyotime, S0053), respectively, according to the manufacturer’s instructions.

#### 2.4.4 Measurement of ferrous iron levels

Ferrous iron levels were measured using a cell ferrous iron colorimetric assay kit (DojinDo Laboratories, VB544 and VB610) according to the manufacturer’s instructions, and the results were examined under a microscope (Olympus Corporation) after FerroOrange (Dojindo, F374) and Hoechst (Beyotime, C1028) staining.

#### 2.4.5 Intracellular reactive oxygen species measurement

Reactive oxygen species (ROS) levels were determined using H2DCFDA (Beyotime, S0033S) and Hoechst (Beyotime, C1028) staining according to the manufacturer’s instructions and subsequent examination under a microscope (Olympus Corporation).

#### 2.4.6 Evaluation of MMP and mPTP

MMP and mPTP were evaluated using JC-1 (BestBio, BB-4105) and BBcellProbe M61 (BestBio, BB-48122), respectively, according to the kits’ instructions. The relative fluorescence intensity of mPTP (Ex = 488 nm; Em = 558 nm) and MMP (530/580 and 485/530 nm) were measured using a Cytomics FC 500 flow cytometer (Agilent).

#### 2.4.7 Mitochondrial ultrastructure assessment

Following treatment, the cells were collected, fixed, washed, dehydrated, embedded, sectioned, stained, and observed using transmission electron microscopy to evaluate mitochondrial ultrastructure.

#### 2.4.8 Western blotting

The concentration of proteins extracted from cells using RIPA lysis buffer (Beyotime, P0013B) was determined using a bicinchoninic acid (BCA) protein assay kit (Beyotime, P0012). Protein expression analysis was performed by WB as described previously (Hu et al., 2023). The target proteins were PTGS2, GPX4, FTMT, and TSPO, and the internal control was β-actin. The quantification and analysis of protein bands were performed using ImageJ 1.5.4 software.

### 2.5 Viruses and plasmids

Adenoviral pAD/FTMT-shRNA (target sense sequence: 5′-GCAAGTGAAGTC TATCAAAGA-3′; antisense sequence: 5′-TCT​TTG​ATA​GAC​TTC​ACT​TGC-3′; MOI:100), adenoviral pAD/FTMT (NCBI reference sequence NC_086036.1; MOI:10) and adenoviral pAD/TSPO (NCBI reference sequence NM_012515.2; MOI:50) were transfected into H9c2 cells cultured in DMEM containing 10% FBS at 37 °C, 5% CO_2_, 21% O_2_, and 95% humidity for 24 h, with 85% transfection efficiency (Supplementary Figure S2).

### 2.6 Quantitative real-time PCR

Total RNA was extracted from H9C2 cells using the Rapid RNA Extraction Kit (ES-Science, RN001). The total RNA was reverse-transcribed into cDNA using a reverse transcription reagent (ES-Science, 11141ES60). cDNA (500 ng) was used in each quantitative real-time PCR (qRT-PCR) reaction and amplified using RT fluorescence quantitative PCR amplification premix. After reaction completion, the mRNA expression levels of the eight genes were determined using the StepOne Plus™ Real-Time PCR System (Thermo Fisher Scientific). The primers are shown in Table 1.

**TABLE 1:** Primers used in quantitative real-time PCR.

Species	Gene	Forward primer	Reverse primer
RAT	HK2	ATG​GAG​TGG​GGA​GCA​TTT​GG	GCC​GCT​GAT​CAT​CTT​CTC​GA
RAT	FTMT	TAA​GTC​CCC​TAC​TGG​CCT​CC	AAG​CCA​TGG​ACA​GGT​ACA​CG
RAT	DLD	TCT​TGG​TTT​GCA​TCG​GTC​GA	GAC​CAG​CAA​CCA​CAT​CTC​CA
RAT	GPD2	GTG​GCG​TAC​GAT​ACC​TCC​AG	CAC​GTT​CAT​GAA​GGG​CCT​CT
RAT	YME1L1	GTT​AAA​GGG​GTG​GAG​GAG​GC	ACA​TCA​GCT​TCT​CCT​GCC​AC
RAT	HIF1-A	GCT​TTA​ACT​TTG​CTG​GCC​CC	TTT​TCG​TTG​GGT​GAG​GGG​AG
RAT	NFS1	TGT​CCT​CAG​AGC​CAT​CGG​TA	TCA​ATG​CCA​TCC​TGC​ACC​AT
RAT	ATP5F1B	GCT​CTG​ACT​GGT​CTG​ACT​GTT​GC	CCT​GGG​TGA​AGC​GGA​AGA​TGT​TG
RAT	TSPO	CCGCCTCGCTGGACACTC	ACC​ATA​GCC​TCC​TCT​GTG​AAA​CC

### 2.7 Bioinformatics

A total of 616 GA-related target genes were obtained from SwissTargetPrediction, the similarity ensemble approach (SEA), SuperPred, CODD-Pred, and GeneCards. 486 Mitochondria-associated genes and 1,517 ferroptosis-associated genes were derived from Gene Set Enrichment Analysis (GSEA). 5,000 Diabetes-related genes and 5,000 heart-related genes were obtained from GeneCards (Supplementary Figure S1). The two-dimensional (2D) structure and three-dimensional (3D) conformer data of GA were obtained from the PubChem database, and the 3D structural data of TSPO was obtained from the AlphaFold database. The simulated docking of molecules and docking scores were accomplished through https://cadd.labshare.cn/.

### 2.8 Statistical analysis

Data are expressed as the mean ± standard deviation and were analyzed using one-way analysis of variance and Tukey’s post hoc test using GraphPad Prism (8.4.2). For the few cases where the parametric test assumptions were violated, the Kruskal-Wallis test with Dunn’s post-hoc test was employed for verification (Supplementary Table S1). A P-value of <0.05 denoted statistical significance.

## 3 Results

### 3.1 GA attenuates cardiac injury and improves cardiac function in T2D rats

**FIGURE 1:**
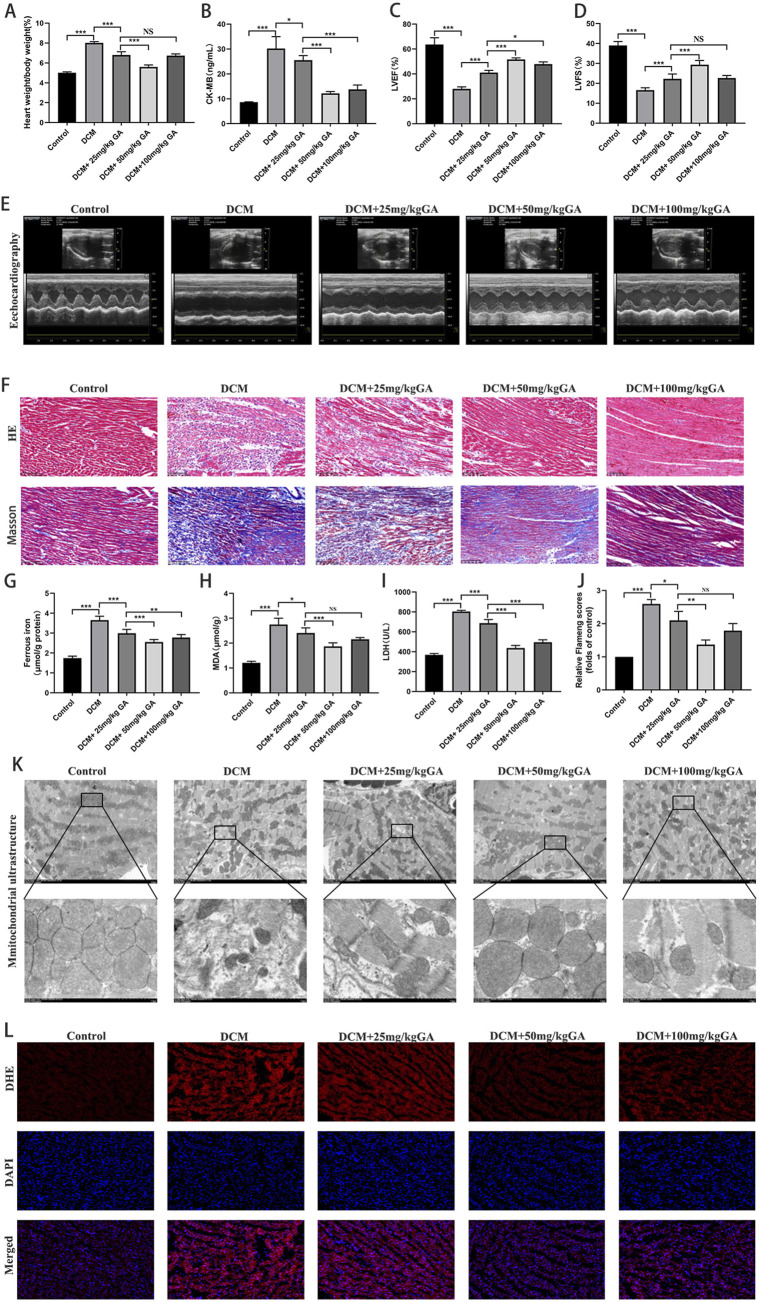
Gallic acid attenuates cardiac injury and improves cardiac function. Type 2 diabetic Rats were treated with low (25 mg/kg), medium (50 mg/kg), or high (100 mg/kg) doses of Gallic acid for 4 weeks. Key parameters of cardiac injury and function were assessed. **(A)** Heart weight to body weight ratio (HW/BW). **(B)** Serum levels of creatine kinase-MB (CK-MB). **(C)** Left ventricular ejection fraction (LVEF). **(D)** Left ventricular fractional shortening (LVFS). **(E)** Representative echocardiographic images. **(F)** Representative images of myocardial tissue sections stained with Hematoxylin and Eosin (H&E) and Masson’s trichrome. **(G)** Myocardial ferrous iron levels. **(H)** Myocardial malondialdehyde (MDA) levels. **(I)** Serum lactate dehydrogenase (LDH) activities. **(J,K)** Representative transmission electron microscopy images and relative Flameng scores of the rat myocardium. **(L)** Representative images of dihydroethidium (DHE) stained myocardium of rat (magnification, ×200). Data are expressed as the mean ± SD (n = 3 or 6). NS, no signiffcance. *P < 0.05, ***P < 0.001, compared with the indicated groups.

The hearts of type 2 diabetic rats were significantly enlarged, and serum levels of CK-MB were markedly elevated (Figures 1A,B). The significant reductions in LVEFs and LVFS rates demonstrated impaired cardiac function (Figures 1C–E). Histopathological examination of H&E and Masson-stained tissue further revealed the characteristic tissue damage of diabetic cardiomyopathy, including marked myocardial fibrosis and structural destruction of cardiomyocytes (Figure 1F). Gavage with low, medium, and high GA concentrations significantly ameliorated these pathological manifestations and effectively attenuated the enzymatic changes, dysfunction, and histopathological damage associated with diabetic cardiomyopathy. Notably, 50 mg/kg GA treatment was the most effective.

LDH, MDA, ferrous iron, and ROS levels were measured in rat myocardial tissue and sera. The results showed that gavage with low, medium, and high GA concentrations significantly ameliorated pathological elevations in ferrous iron (Figure 1G), MDA (Figure 1H), LDH (Figure 1I), and ROS concentrations (Figure 1L) in rats. The middle GA concentration showed the best effect. Transmission electron microscopy showed that the mitochondrial ultrastructure was protected by low, medium, and high concentrations of GA (Figures 1J,K).

### 3.2 GA attenuates high glucose plus palmitate-induced H9C2 cell damage

**FIGURE 2:**
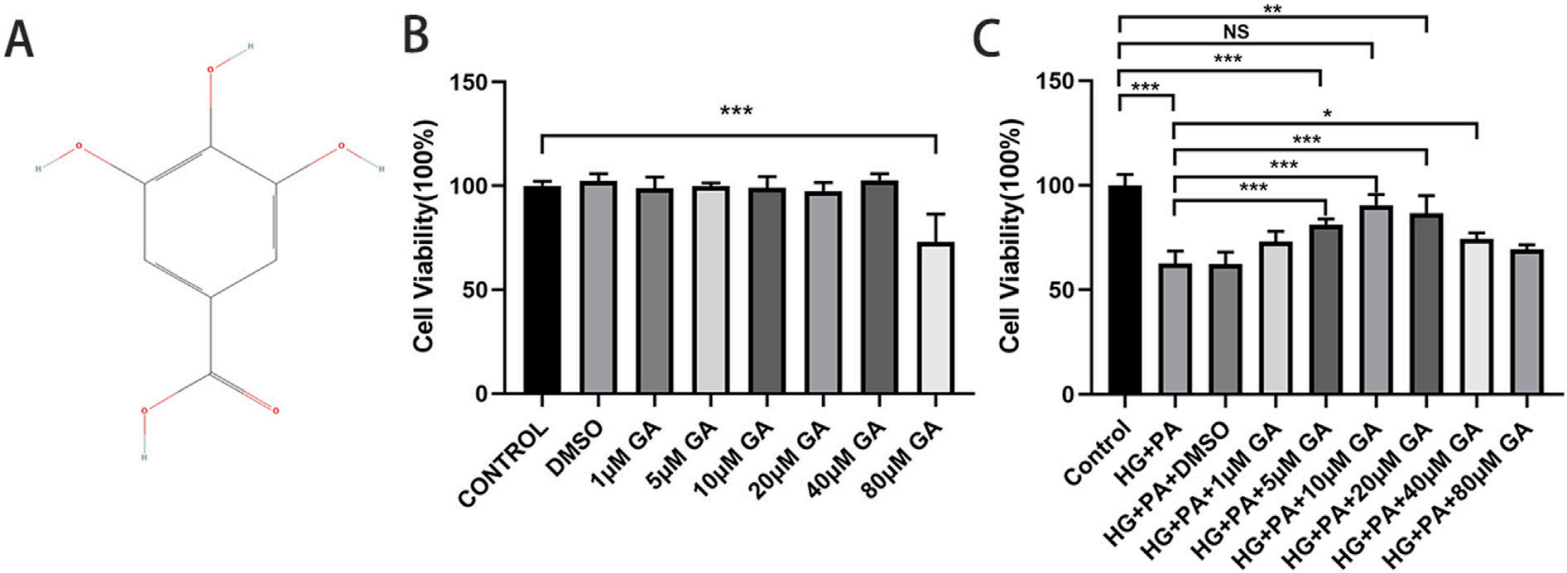
Gallic acid attenuates high glucose plus palmitate-induced H9C2 cell damage. **(A)** The chemical structure of Gallic acid. **(B)** Histogram of CCK-8 detected the cell viability of H9C2 cells pretreated with different concentrations of Gallic acid. **(C)** Histogram of CCK-8 detected the cell viability of normal and HG + PA-injured H9C2 cells pretreated with different concentrations of Gallic acid. Data are expressed as the mean ± SD (n = 3 or 6). *P < 0.05, ***P < 0.001, compared with the indicated groups.

Figure 2A shows the 2D chemical structure of GA. We used the CCK-8 assay to assess the cell viability of normal and HG + PA-injured H9C2 cells pretreated with different concentrations of GA to evaluate its protective effects on the myocardium. No significant decrease in the viability of H9C2 cells treated with ≤40 μM GA was seen, suggesting that ≤40 μM of GA was not significantly cytotoxic (Figure 2B). However, at concentrations of 10–40 μM, GA significantly increased the survival of H9C2 cells subjected to HG + PA damage, indicating that 10–40 μM GA had a protective effect (Figure 2C). Furthermore, at concentrations ranging from 10 to 80 μM, GA significantly inhibited the TSPO upregulation and FTMT downregulation induced by HG + PA damage (Supplementary Figure S3A,B). In subsequent experiments, cells were pretreated with 10 μM GA.

### 3.3 TSPO is a potential target for myocardial protection by GA

**FIGURE 3:**
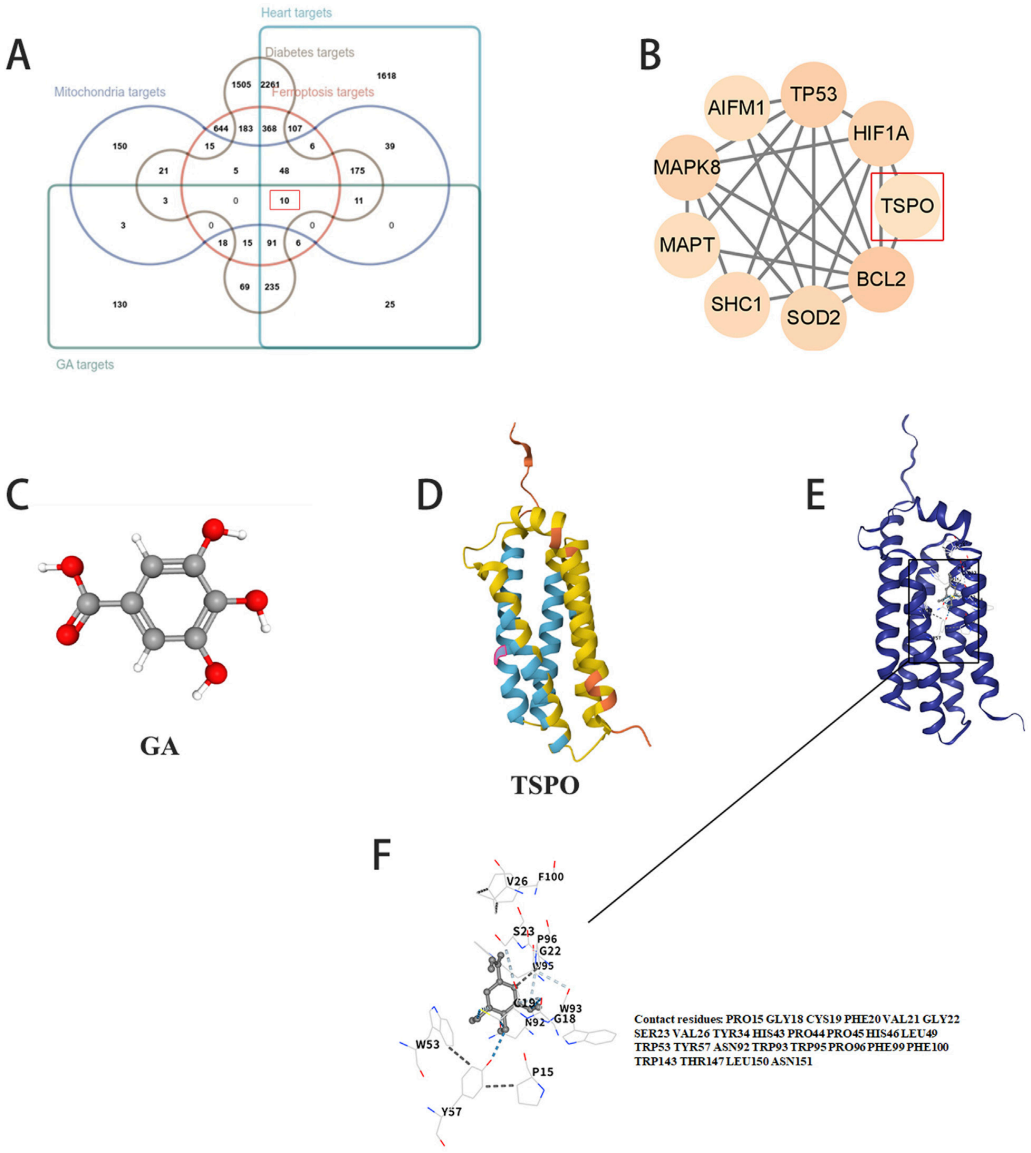
TSPO is a potential target for myocardial protection by gallic acid. **(A)** Gallic acid predicted target genes, ferroptosis related genes, mitochondria related genes with heart related genes, diabetes related genes were taken intersection to obtain 10 genes. **(B)** Intersection gene. **(C)** Chemical structure model of gallic acid. **(D)** Chemical structure model of TSPO. **(E)** Molecular Modeling of gallic acid and TSPO Docking. **(F)** Binding site for gallic acid.

Ten genes were identified at the intersection of predicted GA target genes with ferroptosis-related genes, mitochondria-related genes, heart-related genes, and diabetes-related genes (Figure 3A). The genes are shown in Figure 3B. After reading the relevant literature (Selvaraj and Stocco, 2015; Rupprecht et al., 2022; Flynn and Melov, 2013; Ruvolo et al., 2001; Wischhof et al., 2022; Zhang Y. et al., 2023; Jelinek et al., 2021; Yoshida and Goedert, 2012), TSPO was selected for the next step of the study. The chemical structure model of GA is shown in Figure 3C, and the chemical structure model of TSPO is shown in Figure 3D.

Molecular docking showed that GA could bind to the PRO15, GLY18, CYS19, PHE20, VAL21, GLY22, SER23, VAL26, TYR34, HIS43, PRO44, PRO45, HIS46, LEU49, TRP53, TYR57, ASN92, TRP93, TRP95, PRO96, PHE99, PHE100, TRP143, THR147, LEU150, and ASN151 residues of TSPO (Figures 3E,F).

### 3.4 GA attenuates high glucose plus palmitate-induced ferroptosis in H9C2 cells

**FIGURE 4:**
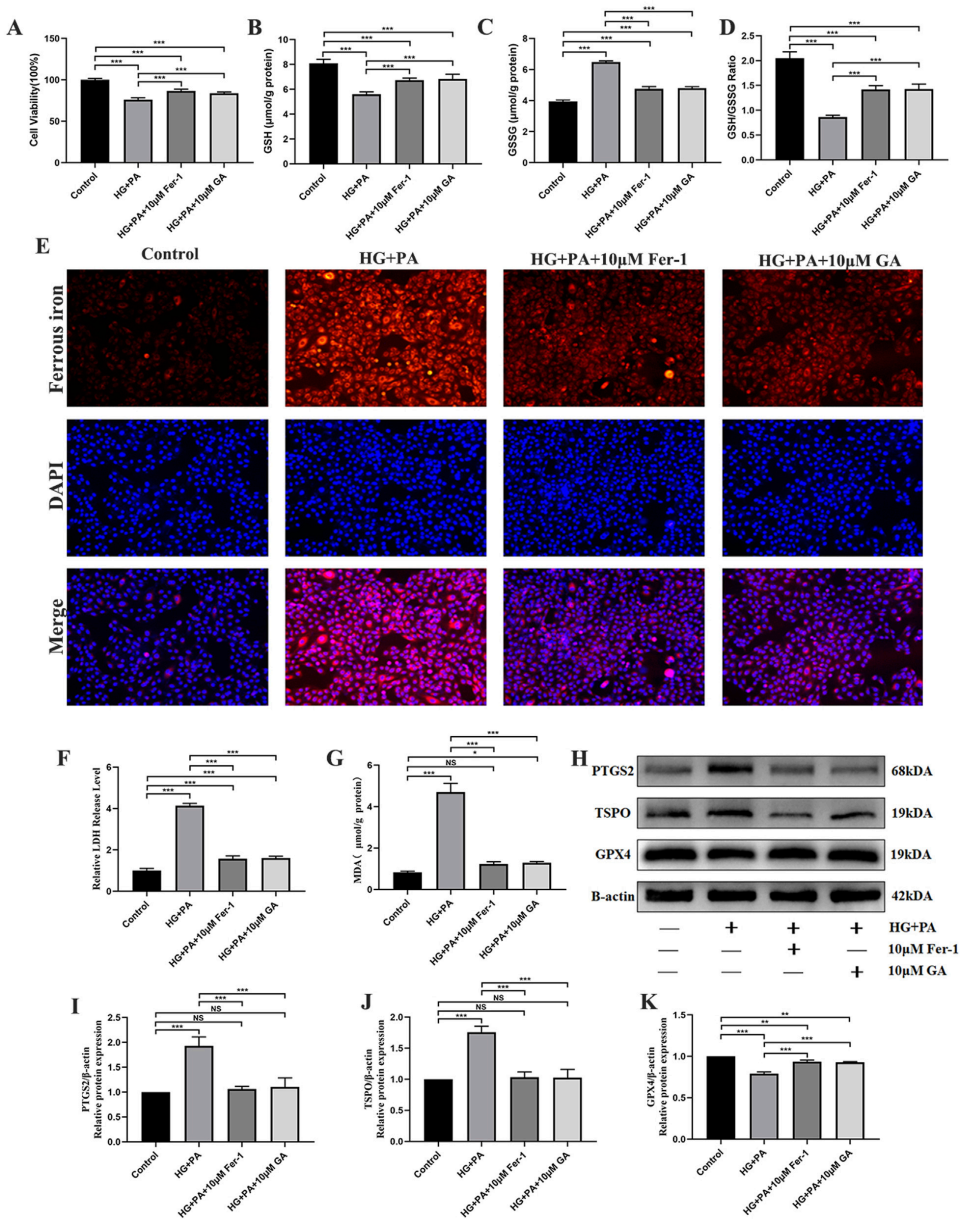
Gallic acid attenuates high glucose plus palmitate-induced ferroptosis in H9C2 cells. Evaluate changes in H9C2 cells exposed to HG + PA following treatment with Fer-1 or gallic acid. **(A)** Cell viability levels. **(B)** Intracellular glutathione (GSH) levels. **(C)** Intracellular oxidized glutathione (GSSG) levels. **(D)** The GSH/GSSG ratio. **(E)** Representative fluorescent images and quantitative analysis of intracellular ferrous iron levels detected by FerroOrange probe. **(F)** Lactate dehydrogenase (LDH) release in the culture medium. **(G)** Malondialdehyde (MDA) levels. **(H)** Representative Western blot bands of prostaglandin-endoperoxide synthase 2 (PTGS2), glutathione peroxidase 4 (GPX4), and translocator protein (TSPO). **(I–K)** Quantitative densitometric analysis of PTGS2, GPX4, and TSPO protein expression levels normalized to β-actin. Data are expressed as the mean ± SD (n = 3 or 6). *P < 0.05, ***P < 0.001, compared with the indicated groups.

The study results showed that HG + PA decreased cell viability, which was attenuated by treatment with Fer-1 (a ferroptosis inhibitor) and GA (Figure 4A). Studies have shown that ferroptosis is involved in DCM (Zhao et al., 2023; Chen et al., 2024), which is consistent with the present study results. In the HG + PA group, the critical indicators of ferroptosis-associated damage (MDA, ferrous iron, GSSG, LDH, and ROS) and the expression of the related protein PTGS2 were significantly increased, whereas the critical indicators of ferroptosis-associated inhibition (GSH and GSH/GSSG) and the expression of the related protein GPX4 were significantly decreased (Figures 4B–K). Of note, GA and Fer-1 similarly inhibited these changes (Figures 4B–K), suggesting that GA prevents high glucose plus palmitate damage in H9C2 cells by inhibiting ferroptosis. In addition, the WB results showed a significant increase in TSPO expression levels in the HG + PA group compared with the control group, while GA and Fer-1 attenuated this change (Figure 4J). Furthermore, unlike Z-VAD (apoptosis inhibitor), GA did not exhibit significant inhibition of HG + PA-induced apoptosis (Supplementary Figure S5).

### 3.5 GA attenuates erastin-induced ferroptosis in H9C2 cells

**FIGURE 5:**
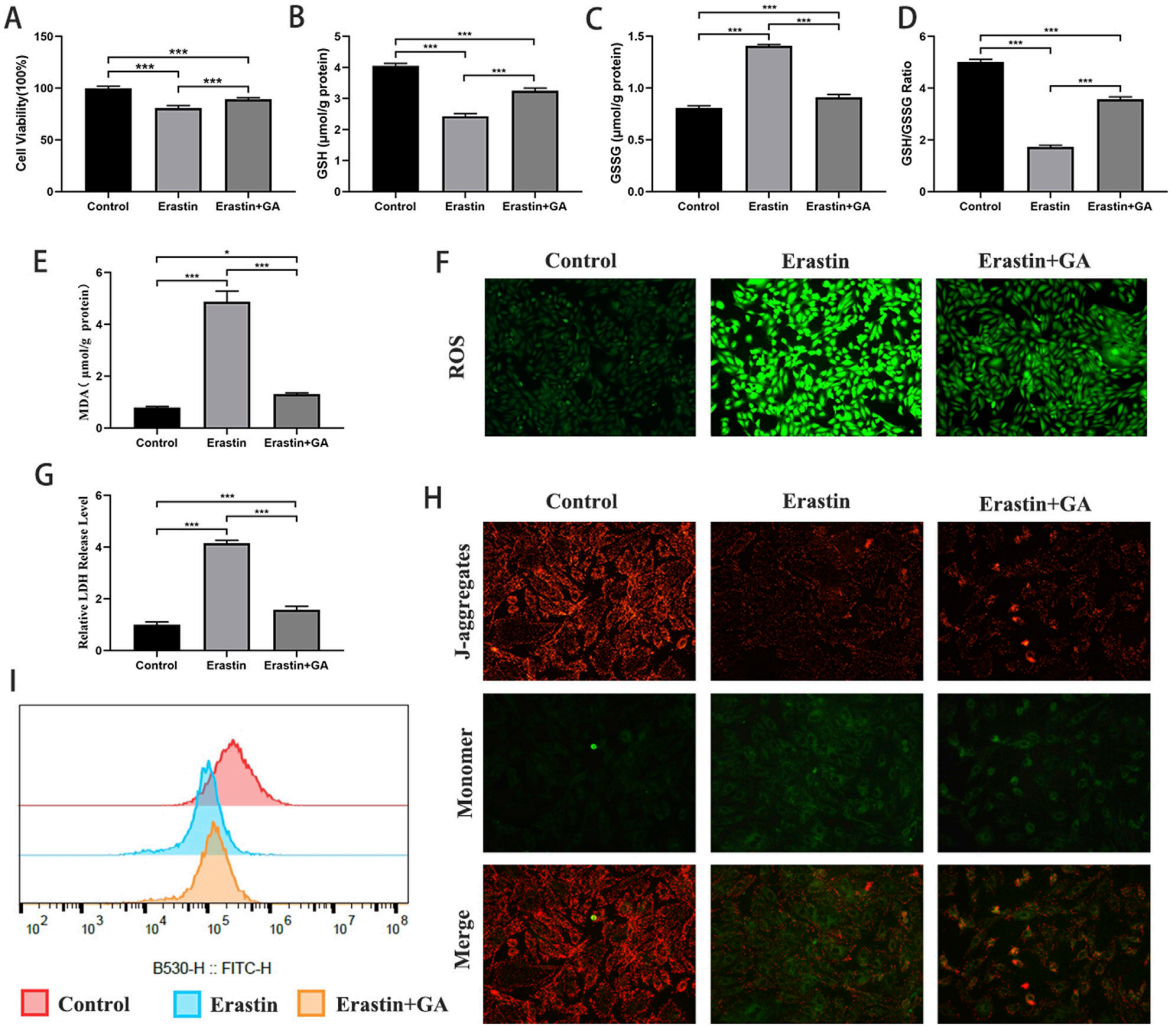
Gallic acid attenuates erastin-induced ferroptosis in H9C2 cells **(A)** Histogram of CCK-8 detected the cell viability of normal and erastin-injured H9C2 cells pretreated with erastin and Gallic acid. **(B–D)** The level of intracellular glutathione (GSH), intracellular oxidized glutathione (GSSG) and the GSH/GSSG ratio. **(E)** Histogram of malondialdehyde level. **(F)** Detection of ROS via DCFH-DA stained (magnification, ×200). **(G)** Histogram of relative LDH level. **(H)** Detection of MMP. **(I)** Detection of mPTP openness by flow cytometry. Data are expressed as the mean ± SD (n = 3 or 6). ***P < 0.001, compared with the indicated groups.

The inhibitory effect of GA against ferroptosis was investigated by establishing an erastin-induced ferroptosis H9C2 cell model. As shown in Figures 5A–G, cell viability, GSH levels, and the GSH/GSSG ratio were significantly decreased, and MDA, GSSG, LDH, and ROS levels were significantly increased in erastin-treated cells. GA significantly inhibited these changes. These findings suggest that ferroptosis occurs in H9C2 cells under erastin induction and that GA can inhibit erastin-induced ferroptosis. The evaluation of MMP and mPTP showed that GA attenuated erastin-induced mitochondrial damage in H9C2 cells (Figures 5H,I).

### 3.6 FTMT mRNA levels are decreased in high glucose plus palmitate-injured and TSPO-overexpressing H9C2 cells

**FIGURE 6:**
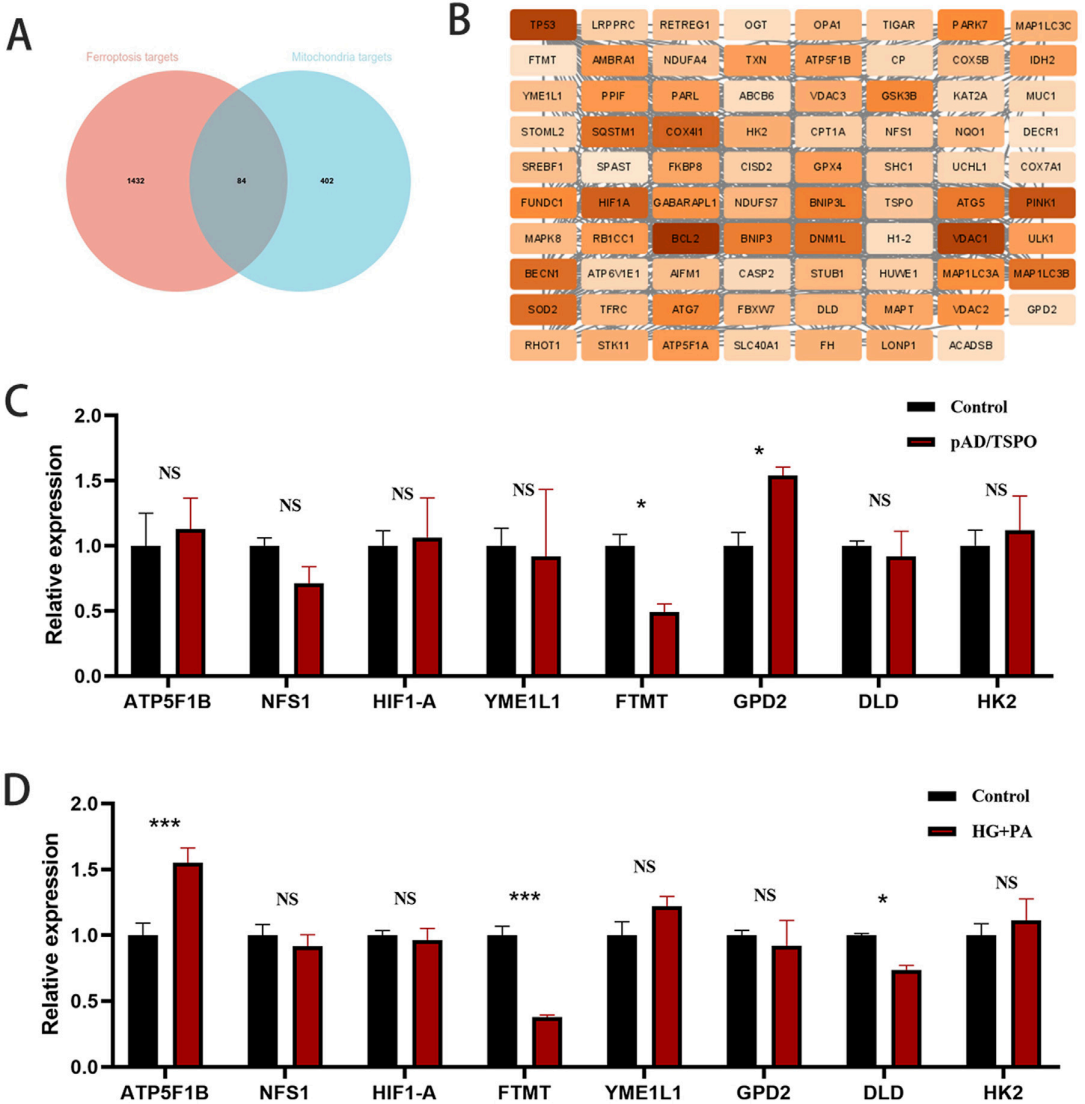
FTMT mRNA levels were decreased in high glucose plus palmitate-injured and TSPO overexpressing H9C2 cells. **(A,B)** Ferroptosis related genes and mitochondria related genes were taken to intersect to obtain 84 genes. **(C)** mRNA expression level of 8 selected genes by qPCR in TSPO overexpression H9c2 cells. **(D)** mRNA expression level 8 selected genes by qPCR in HG + PA treated H9c2 cells. Data are expressed as the mean ± SD (n = 3 or 6). *P < 0.05, ***P < 0.001, compared with the indicated groups. FTMT, mitochondrial ferritin.

The intersection of ferroptosis-related genes and mitochondria-related genes yielded 84 genes (Figures 6A,B). After reading the relevant literature, eight genes were selected and verified by qPCR. mRNA levels of FTMT were significantly decreased in the TSPO-overexpression group compared to the control group, and GPD2 mRNA levels were significantly increased (Figure 6C). The mRNA levels of FTMT and DLD were significantly decreased in the HB + PA group compared to the control group, and the mRNA levels of ATP5F1B were significantly increased (Figure 6D). FTMT mRNA levels were significantly decreased in high glucose plus palmitate-injured and TSPO-overexpressing H9C2 cells compared to controls.

### 3.7 GA attenuates high glucose plus palmitate-induced H9C2 cell damage through TSPO and FTMT

**FIGURE 7:**
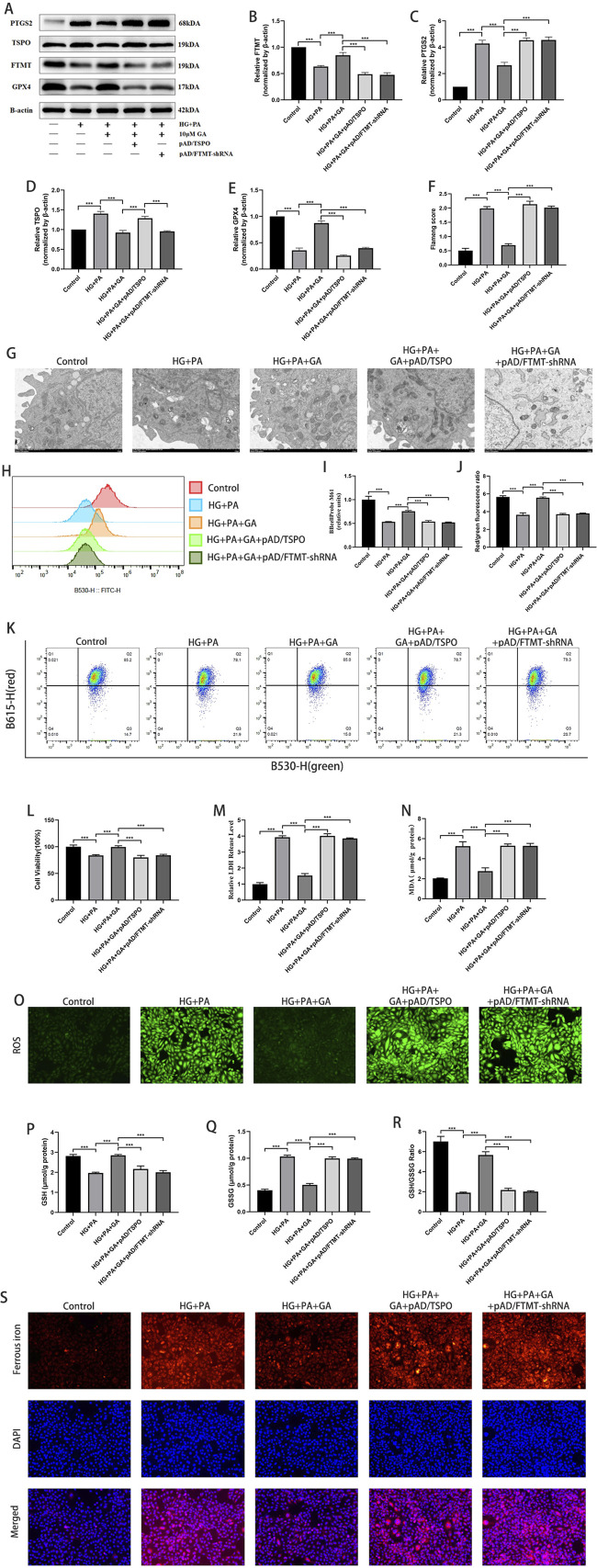
Gallic acid attenuates high glucose plus palmitate-induced H9C2 cell damage through TSPO and FTMT. H9c2 cells were transfected with pAD/TSPO (to overexpress TSPO) or pAD/FTMT-shRNA (to knock down FTMT) prior to HG + PA injury and Gallic acid treatment. **(A)** Representative Western blot bands of PTGS2, TSPO, FTMT, and GPX4. **(B–E)** Quantitative densitometric analysis of FTMT, PTGS2, TSPO, and GPX4 protein expression levels normalized to β-actin. **(F,G)** Representative transmission electron microscopy images and relative flameng scores of H9C2 cells. **(H,I)** Detection of mPTP openness by flow cytometry. **(J,K)** MMP levels were visualized using flow cytometry and expressed as the fluorescence ratio in the upper and lower right quadrants. **(L)** Histogram of CCK-8 detected the cell viability. **(M)** Histogram of relative LDH level. **(N)** Histogram of relative MDA level **(O)** detection of ROS via DCFH--DA stained (magnification, ×200). **(P–R)** Histogram of the level of GSH, GSSG and the GSH/GSSG ratio. **(S)** Intracellular Fe2+ which were revealed by FerroOrange in H9c2 cells. (magnification, ×200). Data are expressed as the mean ± SD (n = 3 or 6). *P < 0.05, ***P < 0.001, compared with the indicated groups.

pAD/TSPO and pAD/FTMT-shRNA were used to verify whether TSPO and FTMT are involved in the attenuation of HG + PA injury-induced ferroptosis by GA. TSPO expression was significantly increased, and FTMT expression was significantly decreased after the addition of pAD/TSPO (Figures 7A,B,D), indicating that the effect of pAD/TSPO was effective and could regulate the protein expression of FTMT. After adding pAD/FTMT-shRNA, the expression of FTMT decreased significantly, indicating that pAD/FTMT-shRNA was effective. Several reports have indicated that mitochondrial dysfunction is involved in DCM (Peng et al., 2023; Cai et al., 2024) and that TSPO (Musman et al., 2017; Fu et al., 2020) and FTMT (Wang P. et al., 2022) are essential for maintaining myocardial mitochondrial homeostasis. In this study, mPTP flow cytometry, MMP flow cytometry, and mitochondrial submicroscopic structural observations were performed. GA prevented high glucose plus palmitate-induced mPTP openings, MMP level increases, and cellular mitochondrial crumpling, twisting, and ridge reductions in H9C2 cells (Figures 7F–K). Meanwhile, pAD/TSPO and pAD/FTMT-shRNA inhibited these changes.

Several studies have indicated a strong association of TSPO (Zhang D. et al., 2023; Bi et al., 2025) and FTMT (Fuhrmann et al., 2020; Wang et al., 2021) with ferroptosis, which is consistent with the results of this study. GA pretreatment significantly prevented increases in PTGS2, LDH, MDA, ferrous iron, ROS, and GSSG levels and decreases in GPX4 levels, cell viability, GSH levels, and the GSH/GSSG ratio induced by HG + PA, whereas pAD/TSPO and pAD/FTMT-shRNA inhibited these changes (Figures 7C,E,L–S). Treatment with pAD/FTMT significantly attenuated the HG + PA-induced increase in PTGS2 protein levels, as well as the decrease in GPX4 protein levels and cell viability. It also markedly reduced mPTP opening in H9C2 cells (Supplementary Figures S4A-G). Furthermore, GA did not exhibit a significant effect on FTL and FTH (Supplementary Figures S6A-G).

## 4 Discussion

Cardiovascular disease is the leading cause of mortality in patients with T2DM, and the development of DCM plays an important role in driving this (Lala et al., 2024; Wong and Sattar, 2023). With the rising prevalence of obesity and an aging population, the prevalence of T2DM is increasing, and the incidence and mortality of DCM are rising rapidly (Zhong et al., 2025). However, the pathogenesis of DCM has not been fully clarified, and there is no treatment for DCM (Tan et al., 2020; Heather et al., 2024). GA is a phenolic acid found in a wide variety of foods and herbs with a wide range of pharmacological activities (Locatelli et al., 2013; Lin et al., 2020). Previous studies have indicated that GA ameliorates endothelial dysfunction and hypotension in diabetic rats, as well as myocardial hypertrophy and left ventricular dysfunction following cardiac ischemia-reperfusion injury (Ramezani Ali Akbari et al., 2019; Ramezani-Aliakbari et al., 2017). Commensurate with these observations, our results indicate that GA effectively attenuated myocardial damage in diabetic cardiomyopathy.

Ferroptosis is a type of regulated cell death. that, unlike autophagy, necrosis, and apoptosis, is primarily characterized by the excessive accumulation of ferrous iron and lipid hydroperoxides (Jiang et al., 2021). Increasing attention has been paid to the involvement of ferroptosis in the pathology of DCM (Zhao et al., 2023), and some natural plant products have been shown to inhibit ferroptosis and alleviate myocardial damage in DCM (Wang X. et al., 2022; Wu et al., 2024). Ferroptosis is closely interrelated with mitochondria (Glover et al., 2024; Dixon and Olzmann, 2024). Mitochondria are key organelles for the survival and normal function of cardiomyocytes, which are energy-intensive cells. However, the structure and function of mitochondria are impaired under hyperglycemic conditions, leading to disturbances in energy metabolism, ROS accumulation, increased oxidative stress, and the dysregulation of calcium homeostasis, which ultimately triggers cardiomyocyte hypertrophy, fibrosis, and microvascular pathology, contributing to the development and progression of DCM (Cai et al., 2022). Numerous studies have demonstrated that mitochondria may be a therapeutic target for DCM (Cai et al., 2022; Bhagani et al., 2020). In this study, GA was shown to significantly reduce ferrous ion accumulation and PTGS2 expression, increase GPX4 expression, inhibit excessive ROS production, and attenuate lipid metabolism disorders. The mitochondrial ultrastructural changes and altered mPTP and MMP levels demonstrated the protective effect of GA on mitochondrial homeostasis. It is worth noting that, unlike the apoptosis inhibitor Z-VAD, GA did not significantly suppress HG + PA-induced apoptosis, as evidenced by the lack of a marked change in Caspase-3 activity. This preliminary finding suggests that the protective effect of GA may be dissociated from the apoptotic pathway and appears to be mediated primarily through the inhibition of ferroptosis.

Multiple gene sets were intersected to explore the possible targets through which GA attenuates ferroptosis and maintains mitochondrial homeostasis in diabetic cardiomyopathy. BCL2, SOD2, SHC1, MAPT, MAPK8, AIFM1, TP53, HIF1A, and TSPO were identified. BCL2 is a classical mitochondrial outer membrane protein that regulates cell death and inhibits apoptosis in a variety of cells by modulating mitochondrial membrane permeability (Ruvolo et al., 2001; Eguchi et al., 1992). Some studies also reported that it inhibited autophagy (Strappazzon et al., 2011; Chen et al., 2010). SOD2 is a mitochondrial protein that generates superoxide anion radicals in cells (Flynn and Melov, 2013). SHC1 has three main isoforms that differ in activity and subcellular location. The longest isoform (p66Shc) may be involved in regulating lifespan and ROS, the p46Shc isoform targets the mitochondrial matrix, and all three isoforms are junction proteins in signal transduction pathways (Jelinek et al., 2021). MAPT is a microtubule-associated protein that promotes microtubule assembly and stability and may be involved in the establishment and maintenance of neuronal polarity (Yoshida and Goedert, 2012). MAPK8 is a member of the MAP kinase family. MAP kinases act as integration points for a wide range of biochemical signals and are involved in cellular processes such as proliferation, differentiation, transcriptional regulation, and development (Zhang Y. et al., 2023). AIFM1 functions as a NADH oxidoreductase and apoptosis regulator, frequently found in the mitochondrial membrane space (Wischhof et al., 2022). TP53 responds to different cellular stresses to regulate the expression of target genes to induce cell cycle arrest, apoptosis, senescence, DNA repair or metabolic changes. Mutations in this gene have been associated with a number of human cancers (Wischhof et al., 2022). HIF1A is a master transcriptional regulator of the adaptive response to hypoxia and activates the transcription of many genes, including genes involved in energy metabolism, angiogenesis, and apoptosis (Prabhakar and Semenza, 2012). TSPO is a mitochondrial membrane protein involved in the regulation of cellular functions such as ferroptosis (Zhang D. et al., 2023), cellular respiration and oxidative processes (Baglini et al., 2024), protein import, and ion transport (Baglini et al., 2024). TSPO plays a role in regulating cardiac physiological processes, and several studies have shown that TSPO ligands have different cardiac-modulating effects. TSPO ligands Ro 5-4864 and PK 11195 reduce calcium currents, thereby decreasing sarcoplasmic reticulum calcium (Bolger et al., 1990). PK 11195 (a TSPO ligand) has also been shown to antagonize calcium channel blockers in guinea pig myocardium (Mestre et al., 1985). A TSPO ligand was also shown to play a protective role in heart disease by reducing ROS generation and preventing mitochondrial dysfunction and stress-dependent cardiomyocyte loss (Baglini et al., 2024). TSPO was also reported to inhibit oxidative stress and maintain mitochondrial homeostasis by directly down-regulating TSPO expression to alleviate myocardial hypoxia-enriched oxygen injury (Meng et al., 2020). TSPO also affects the activity of other mitochondrial components, such as mPTP, VDAC, and IMAC, which play a critical role in acute and chronic cardiac diseases (Baglini et al., 2024). The WB results showed that GA significantly reduced the expression of TSPO, and the inhibition of ferroptosis and the maintenance of mitochondrial homeostasis by GA were prevented after the overexpression of TSPO using pAD/TSPO, suggesting that the protective effect of GA was achieved by regulating TSPO.

The qRT-PCR results indicated that the mRNA levels of FTMT were significantly decreased in H9C2 cells in both HG + PA and TSPO overexpression settings. The WB results further showed that the protein level of FTMT decreased significantly. FTMT is an iron storage protein located in mitochondria and highly expressed in the heart. It catalyzes the oxidation of ferrous iron to trivalent iron and stores iron in a soluble, non-toxic form (Yanatori et al., 2023). Previous studies reported that FTMT deficiency enhanced ferroptosis and exacerbated ventricular tachyarrhythmias after myocardial infarction in mice (Chang et al., 2025). Mice lacking FTMT were also reported to be more sensitive to adriamycin-mediated cardiotoxicity and have aggravated myocardial mitochondrial damage (Maccarinelli et al., 2014). Meanwhile, the overexpression of FTMT significantly attenuated cerebral ischemia-reperfusion injury-induced ferroptosis and subsequent brain damage in mice (Wang et al., 2021). In addition, FTMT overexpression rescued oxygen and glucose deprivation and reperfusion-induced mitochondrial iron overload and mitochondrial dysfunction in neuronal cells (Wang P. et al., 2022). Consistent with the above studies, the present study showed that the effect of GA in inhibiting ferroptosis and maintaining mitochondrial homeostasis was prevented after silencing FTMT with pAD/FTMT-shRNA. To further investigate iron partitioning, we assessed ferritin in the cytoplasm. Our findings revealed that GA does not affect FTL and FTH levels but effectively regulates FTMT and ferrous iron content in mitochondria. This discovery further confirms that its protective mechanism primarily targets mitochondrial iron dysregulation.

While our results demonstrate a clear regulatory relationship between TSPO overexpression and the downregulation of FTMT, we acknowledge that the precise molecular mechanism remains unclear and requires further elucidation. Based on existing literature and the roles of both proteins in mitochondrial iron and redox homeostasis, we propose several plausible mechanisms that might underlie this regulation. TSPO, as a mitochondrial outer membrane protein involved in cholesterol transport, ROS modulation, and apoptosis regulation, may influence FTMT expression through indirect transcriptional pathways. For instance, TSPO has been shown to modulate reactive oxygen species (ROS) generation and mitochondrial membrane permeability, which could subsequently affect transcription factors such as NRF2 or NF-κB (Zhang D. et al., 2023; Li et al., 2024), known regulators of iron metabolism and ferroptosis-related genes including FTMT (Song et al., 2024; Yang et al., 2015). Alternatively, TSPO might participate in protein–protein interactions with transporters or chaperones involved in iron trafficking, thereby altering iron availability in the mitochondrial matrix and indirectly suppressing FTMT expression. Another possibility involves the alteration of mitochondrial iron handling pathways—TSPO overexpression may enhance iron export or compartmentalization, reducing the regulatory demand for FTMT-mediated iron storage. Despite these hypotheses, the precise regulatory mechanism—whether TSPO directly controls FTMT expression or acts through intermediate signaling pathways—remains unclear and warrants future targeted studies. Furthermore, the use of H9C2 cells, while common, may not fully recapitulate adult cardiomyocyte biology. Moreover, the absence of human samples or clinical data restricts the translational relevance of our findings.

## 5 Conclusion

In conclusion, GA pretreatment attenuated diabetic cardiomyopathy by downregulating TSPO expression, which subsequently promoted FTMT upregulation. This TSPO/FTMT pathway axis reduced myocardial ferroptosis, attenuated intracellular ROS accumulation, inhibited lipid metabolism dysregulation, and preserved mitochondrial homeostasis.

## Data Availability

The original contributions presented in the study are included in the article/Supplementary Material, further inquiries can be directed to the corresponding authors.

## Supplementary material

SUPPLEMENTARY FIGURE S1 Histogram of CCK-8 detected the cell viability of H9C2 cells pretreated with different concentrations of palmitate. Data are expressed as the mean ± SD (n = 6). NS, no signiffcance. *P < 0.05, ***P < 0.001, compared with the indicated groups.

SUPPLEMENTARY FIGURE S2 Preliminary Experiment on Adenovirus Infection Efficiency. **(A)** Detection of FTMT mRNA levels in H9C2 cells infected with pAD/FTMT adenovirus at different MOIs by qPCR. **(B)** Detection of FTMT mRNA levels in H9C2 cells infected with pAD/FTMT-shRNA adenovirus at different MOIs by qPCR. **(C)** Detection of TSPO mRNA levels in H9C2 cells infected with pAD/TSPO adenovirus at different MOIs by qPCR. Data are expressed as the mean ± SD (n = 6). NS, no signiffcance. **P < 0.01, ***P < 0.001, compared with the indicated groups.

SUPPLEMENTARY FIGURE S3 Effects of different concentrations of gallic acid on TSPO and FTMT mRNA levels in H9C2 cells. **(A)** Detection of FTMT mRNA levels in H9C2 cells pretreated with different concentrations of Gallic acid. **(B)** Detection of TSPO mRNA levels in H9C2 cells pretreated with different concentrations of Gallic acid. Data are expressed as the mean ± SD (n = 5). NS, no signiffcance. **P < 0.01, ***P < 0.001, compared with the indicated groups.

SUPPLEMENTARY FIGURE S4 FTMT overexpression alleviates HG + PA-induced damage. **(A)** Representative Western blot bands of PTGS2, TSPO, FTMT, and GPX4. **(B-E)** Quantitative densitometric analysis of FTMT, PTGS2, TSPO, and GPX4 protein expression levels normalized to β-actin. **(F)** Histogram of CCK-8 detected the cell viability of H9C2 cells pretreated with HG + PA and pAD/ftmt. **(G)** MMP levels were visualized using fluorescence microscope. Data are expressed as the mean ± SD (n = 3 or 6). NS, no signiffcance. **P < 0.01, ***P < 0.001, compared with the indicated groups.

SUPPLEMENTARY FIGURE S5 Capase-3 activity were assayed in H9c2 cells exposed to HG + PA after pretreatment with 10 μM alpinetin or 40 μM Z-VAD. Data are expressed as the mean ± SD (n = 4). NS, no signiffcance. ***P < 0.001, compared with the indicated groups.

SUPPLEMENTARY FIGURE S6 GA did not exhibit a significant effect on FTL and FTH. **(A)** Representative Western blot bands of FTH and FTL. **(B-E)** Quantitative densitometric analysis of FTH and FTL protein expression levels normalized to β-actin. Data are expressed as the mean ± SD (n = 3). NS, no signiffcance. ***P < 0.001, compared with the indicated groups.

## References

[B1] Baglini E. Poggetti V. Cavallini C. Petroni D. Forini F. Nicolini G. (2024). Targeting the translocator protein (18 kDa) in cardiac diseases: state of the art and future opportunities. J. Med. Chem. 67 (1), 17–37. 10.1021/acs.jmedchem.3c01716 38113353 PMC10911791

[B2] Bai J. J. Zhang Y. Tang C. Hou Y. Ai X. Chen X. (2021). Gallic acid: pharmacological activities and molecular mechanisms involved in inflammation-related diseases. Biomed. Pharmacother. 133, 110985. 10.1016/j.biopha.2020.110985 33212373

[B3] Bai X. Yang C. Jiao L. Diao H. Meng Z. Wang L. (2021). LncRNA MIAT impairs cardiac contractile function by acting on mitochondrial translocator protein TSPO in a mouse model of myocardial infarction. Signal Transduct. Target Ther. 6 (1), 172. 10.1038/s41392-021-00538-y 33941765 PMC8093248

[B4] Bhagani H. Nasser S. A. Dakroub A. El-Yazbi A. F. Eid A. A. Kobeissy F. (2020). The mitochondria: a target of polyphenols in the treatment of diabetic cardiomyopathy. Int. J. Mol. Sci. 21 (14), 4962. 10.3390/ijms21144962 32674299 PMC7404043

[B5] Bi T. Zhao Q. Wang T. Huang R. Liu B. Liu X. (2025). Disruption of ferroptosis inhibition and immune evasion with tumor-activatable prodrug for boosted photodynamic/chemotherapy eradication of drug-resistant tumors. Adv. Healthc. Mater 14 (2), e2403473. 10.1002/adhm.202403473 39530628

[B6] Bolger G. T. Abraham S. Oz N. Weissman B. A. (1990). Interactions between peripheral-type benzodiazepine receptor ligands and an activator of voltage-operated calcium channels. Can. J. Physiol. Pharmacol. 68 (1), 40–45. 10.1139/y90-005 1691678

[B7] Bouzghaya S. Amri M. Homblé F. (2020). Improvement of diabetes symptoms and complications by an aqueous extract of Linum usitatissimum (L.) seeds in alloxan-induced diabetic mice. J. Med. Food 23 (10), 1077–1082. 10.1089/jmf.2019.0205 32109173

[B8] Bréhat J. Leick S. Musman J. Su J. B. Eychenne N. Giton F. (2024). Identification of a mechanism promoting mitochondrial sterol accumulation during myocardial ischemia-reperfusion: role of TSPO and STAR. Basic Res. Cardiol. 119 (3), 481–503. 10.1007/s00395-024-01043-3 38517482

[B9] Cai C. Wu F. He J. Zhang Y. Shi N. Peng X. (2022). Mitochondrial quality control in diabetic cardiomyopathy: from molecular mechanisms to therapeutic strategies. Int. J. Biol. Sci. 18 (14), 5276–5290. 10.7150/ijbs.75402 36147470 PMC9461654

[B10] Cai W. Chong K. Huang Y. Huang C. Yin L. (2024). Empagliflozin improves mitochondrial dysfunction in diabetic cardiomyopathy by modulating ketone body metabolism and oxidative stress. Redox Biol. 69, 103010. 10.1016/j.redox.2023.103010 38160540 PMC10792762

[B11] Chang Y. Li S. Chen K. Wang Y. Huang D. Wang X. (2025). Mitochondrial ferritin inhibition aggravates pacing-induced ventricular arrhythmias after myocardial infarction by promoting cardiomyocyte ferroptosis. Cell Signal 131, 111683. 10.1016/j.cellsig.2025.111683 40023300

[B12] Chen H.-M. Wu Y.-C. Chia Y.-C. Chang F. R. Hsu H. K. Hsieh Y. C. (2009). Gallic acid, a major component of Toona sinensis leaf extracts, contains a ROS-mediated anti-cancer activity in human prostate cancer cells. Cancer Lett. 286 (2), 161–171. 10.1016/j.canlet.2009.05.040 19589639

[B13] Chen D. Gao F. Li B. Wang H. Xu Y. Zhu C. (2010). Parkin mono-ubiquitinates Bcl-2 and regulates autophagy. J. Biol. Chem. 285 (49), 38214–38223. 10.1074/jbc.M110.101469 20889974 PMC2992255

[B14] Chen Z. Li S. Liu M. Yin M. Chen J. Li Y. (2024). Nicorandil alleviates cardiac microvascular ferroptosis in diabetic cardiomyopathy: role of the mitochondria-localized AMPK-Parkin-ACSL4 signaling pathway. Pharmacol. Res. 200, 107057. 10.1016/j.phrs.2024.107057 38218357

[B15] Da Dalt L. Cabodevilla A. G. Goldberg I. J. Norata G. D. (2023). Cardiac lipid metabolism, mitochondrial function, and heart failure. Cardiovasc Res. 119 (10), 1905–1914. 10.1093/cvr/cvad100 37392421 PMC10681665

[B16] Das A. Nikhil A. Shiekh P. A. Yadav B. Jagavelu K. Kumar A. (2024). Ameliorating impaired cardiac function in myocardial infarction using exosome-loaded gallic-acid-containing polyurethane scaffolds. Bioact. Mater 33, 324–340. 10.1016/j.bioactmat.2023.11.009 38076649 PMC10701288

[B17] Dixon S. J. Olzmann J. A. (2024). The cell biology of ferroptosis. Nat. Rev. Mol. Cell Biol. 25 (6), 424–442. 10.1038/s41580-024-00703-5 38366038 PMC12187608

[B18] Eguchi Y. Ewert D. L. Tsujimoto Y. (1992). Isolation and characterization of the chicken bcl-2 gene: expression in a variety of tissues including lymphoid and neuronal organs in adult and embryo. Nucleic Acids Res. 20 (16), 4187–4192. 10.1093/nar/20.16.4187 1508712 PMC334124

[B19] Fang X. Ardehali H. Min J. Wang F. (2023). The molecular and metabolic landscape of iron and ferroptosis in cardiovascular disease. Nat. Rev. Cardiol. 20 (1), 7–23. 10.1038/s41569-022-00735-4 35788564 PMC9252571

[B20] Flynn J. M. Melov S. (2013). SOD2 in mitochondrial dysfunction and neurodegeneration. Free Radic. Biol. Med. 62, 4–12. 10.1016/j.freeradbiomed.2013.05.027 23727323 PMC3811078

[B21] Fu Y. Wang D. Wang H. Cai M. Li C. Zhang X. (2020). TSPO deficiency induces mitochondrial dysfunction, leading to hypoxia, angiogenesis, and a growth-promoting metabolic shift toward glycolysis in glioblastoma. Neuro Oncol. 22 (2), 240–252. 10.1093/neuonc/noz183 31563962 PMC7442372

[B22] Fuhrmann D. C. Mondorf A. Beifuß J. Jung M. Brüne B. (2020). Hypoxia inhibits ferritinophagy, increases mitochondrial ferritin, and protects from ferroptosis. Redox Biol. 36, 101670. 10.1016/j.redox.2020.101670 32810738 PMC7452134

[B23] Glover H. L. Schreiner A. Dewson G. Tait S. W. G. (2024). Mitochondria and cell death. Nat. Cell Biol. 26 (9), 1434–1446. 10.1038/s41556-024-01429-4 38902422

[B24] Heather L. C. Gopal K. Srnic N. Ussher J. R. (2024). Redefining diabetic cardiomyopathy: perturbations in substrate metabolism at the heart of its pathology. Diabetes 73 (5), 659–670. 10.2337/dbi23-0019 38387045 PMC11043056

[B25] Hsu P.-S. Liu S.-T. Chiu Y.-L. Tsai C.-S. (2023). The functional role of myogenin in cardiomyoblast H9c2 cells treated with high glucose and palmitic acid: insights into No-Rejection heart transplantation. Int. J. Mol. Sci. 24 (17), 13031. 10.3390/ijms241713031 37685838 PMC10487901

[B26] Hu J. Gu W. Ma N. Fan X. Ci X. (2022). Leonurine alleviates ferroptosis in cisplatin-induced acute kidney injury by activating the Nrf2 signalling pathway. Br. J. Pharmacol. 179 (15), 3991–4009. 10.1111/bph.15834 35303762

[B27] Hu T. Zou H.-X. Le S.-Y. Wang Y. R. Qiao Y. M. Yuan Y. (2023). Tanshinone IIA confers protection against myocardial ischemia/reperfusion injury by inhibiting ferroptosis and apoptosis via VDAC1. Int. J. Mol. Med. 52 (5), 109. 10.3892/ijmm.2023.5312 37800609 PMC10558218

[B28] Jelinek H. F. Helf C. Khalaf K. (2021). Human SHC-transforming protein 1 and its isoforms p66shc: a novel marker for prediabetes. J. Diabetes Investig. 12 (10), 1881–1889. 10.1111/jdi.13551 33759377 PMC8504898

[B29] Jiang X. Stockwell B. R. Conrad M. (2021). Ferroptosis: mechanisms, biology and role in disease. Nat. Rev. Mol. Cell Biol. 22 (4), 266–282. 10.1038/s41580-020-00324-8 33495651 PMC8142022

[B30] Ke D. Zhang Z. Liu J. Chen P. Li J. Sun X. (2023). Ferroptosis, necroptosis and cuproptosis: novel forms of regulated cell death in diabetic cardiomyopathy. Front. Cardiovasc Med. 10, 1135723. 10.3389/fcvm.2023.1135723 36970345 PMC10036800

[B31] Kosuru R. Y. Roy A. Das S. K. Bera S. (2018). Gallic acid and gallates in human health and disease: do mitochondria hold the key to success? Mol. Nutr. Food Res. 62 (1), 1700699. 10.1002/mnfr.201700699 29178387

[B32] Lala A. Mentz R. J. Santos-Gallego C. G. (2024). The quest for understanding diabetic cardiomyopathy: can we preserve function and prevent failure? J. Am. Coll. Cardiol. 84 (2), 149–151. 10.1016/j.jacc.2024.05.036 38888535

[B33] Li J. Jia Y.-C. Ding Y.-X. Bai J. Cao F. Li F. (2023). The crosstalk between ferroptosis and mitochondrial dynamic regulatory networks. Int. J. Biol. Sci. 19 (9), 2756–2771. 10.7150/ijbs.83348 37324946 PMC10266069

[B34] Li Y. Chen L. Sottas C. Raul M. C. Patel N. D. Bijja J. R. (2024). The mitochondrial TSPO ligand atriol mitigates metabolic-associated steatohepatitis by downregulating CXCL1. Metabolism 159, 155942. 10.1016/j.metabol.2024.155942 38871077 PMC11374472

[B35] Lin Y. Luo T. Weng A. Huang X. Yao Y. Fu Z. (2020). Gallic acid alleviates gouty arthritis by inhibiting NLRP3 inflammasome activation and pyroptosis through enhancing Nrf2 signaling. Front. Immunol. 11, 580593. 10.3389/fimmu.2020.580593 33365024 PMC7750458

[B36] Locatelli C. Filippin-Monteiro F. B. Creczynski-Pasa T. B. (2013). Alkyl esters of gallic acid as anticancer agents: a review. Eur. J. Med. Chem. 60, 233–239. 10.1016/j.ejmech.2012.10.056 23291333

[B37] Maccarinelli F. Gammella E. Asperti M. Regoni M. Biasiotto G. Turco E. (2014). Mice lacking mitochondrial ferritin are more sensitive to doxorubicin-mediated cardiotoxicity. J. Mol. Med. Berl. 92 (8), 859–869. 10.1007/s00109-014-1147-0 24728422 PMC4118045

[B38] Meng Y. Tian M. Yin S. Lai S. Zhou Y. Chen J. (2020). Downregulation of TSPO expression inhibits oxidative stress and maintains mitochondrial homeostasis in cardiomyocytes subjected to anoxia/reoxygenation injury. Biomed. Pharmacother. 121, 109588. 10.1016/j.biopha.2019.109588 31707350

[B39] Mestre M. Carriot T. Belin C. Uzan A. Renault C. Dubroeucq M. C. (1985). Electrophysiological and pharmacological evidence that peripheral type benzodiazepine receptors are coupled to calcium channels in the heart. Life Sci. 36 (4), 391–400. 10.1016/0024-3205(85)90126-2 2578209

[B40] Musman J. Paradis S. Panel M. Pons S. Barau C. Caccia C. (2017). A TSPO ligand prevents mitochondrial sterol accumulation and dysfunction during myocardial ischemia-reperfusion in hypercholesterolemic rats. Biochem. Pharmacol. 142, 87–95. 10.1016/j.bcp.2017.06.125 28645478

[B41] Peng C. Zhang Y. Lang X. Zhang Y. (2023). Role of mitochondrial metabolic disorder and immune infiltration in diabetic cardiomyopathy: new insights from bioinformatics analysis. J. Transl. Med. 21 (1), 66. 10.1186/s12967-023-03928-8 36726122 PMC9893675

[B42] Prabhakar N. R. Semenza G. L. (2012). Adaptive and maladaptive cardiorespiratory responses to continuous and intermittent hypoxia mediated by hypoxia-inducible factors 1 and 2. Physiol. Rev. 92 (3), 967–1003. 10.1152/physrev.00030.2011 22811423 PMC3893888

[B43] Ramezani Ali Akbari F. Badavi M. Dianat M. Mard S. A. Ahangarpour A. (2019). Gallic acid improves oxidative stress and inflammation through regulating micrornas expressions in the blood of diabetic rats. Acta Endocrinol. (Buchar). 15 (2), 187–194. 10.4183/aeb.2019.187 31508175 PMC6711635

[B44] Ramezani-Aliakbari F. Badavi M. Dianat M. Mard S. A. Ahangarpour A. (2017). Effects of gallic acid on hemodynamic parameters and infarct size after ischemia-reperfusion in isolated rat hearts with alloxan-induced diabetes. Biomed. Pharmacother. 96, 612–618. 10.1016/j.biopha.2017.10.014 29035826

[B45] Rupprecht R. Wetzel C. H. Dorostkar M. Herms J. Albert N. L. Schwarzbach J. (2022). Translocator protein (18kDa) TSPO: a new diagnostic or therapeutic target for stress-related disorders? Mol. Psychiatry 27 (7), 2918–2926. 10.1038/s41380-022-01561-3 35444254

[B46] Ruvolo P. P. Deng X. May W. S. (2001). Phosphorylation of Bcl2 and regulation of apoptosis. Leukemia 15 (4), 515–522. 10.1038/sj.leu.2402090 11368354

[B47] Saeedi P. Petersohn I. Salpea P. Malanda B. Karuranga S. Unwin N. (2019). Global and regional diabetes prevalence estimates for 2019 and projections for 2030 and 2045: results from the international diabetes Federation diabetes atlas, 9^th^ edition. Diabetes Res. Clin. Pract. 157, 107843. 10.1016/j.diabres.2019.107843 31518657

[B48] Selvaraj V. Stocco D. M. (2015). The changing landscape in translocator protein (TSPO) function. Trends Endocrinol. Metab. 26 (7), 341–348. 10.1016/j.tem.2015.02.007 25801473 PMC7171652

[B49] Shoshan-Barmatz V. Pittala S. Mizrachi D. (2019). VDAC1 and the TSPO: expression, interactions, and associated functions in health and disease states. Int. J. Mol. Sci. 20 (13), 3348. 10.3390/ijms20133348 31288390 PMC6651789

[B50] Song Y. Gao M. Wei B. Huang X. Yang Z. Zou J. (2024). Mitochondrial ferritin alleviates ferroptosis in a kainic acid-induced mouse epilepsy model by regulating iron homeostasis: involvement of nuclear factor erythroid 2-related factor 2. CNS Neurosci. Ther. 30 (3), e14663. 10.1111/cns.14663 38439636 PMC10912846

[B51] Strappazzon F. Vietri-Rudan M. Campello S. Nazio F. Florenzano F. Fimia G. M. (2011). Mitochondrial BCL-2 inhibits AMBRA1-induced autophagy. EMBO J. 30 (7), 1195–1208. 10.1038/emboj.2011.49 21358617 PMC3094111

[B52] Tan Y. Zhang Z. Zheng C. Wintergerst K. A. Keller B. B. Cai L. (2020). Mechanisms of diabetic cardiomyopathy and potential therapeutic strategies: preclinical and clinical evidence. Nat. Rev. Cardiol. 17 (9), 585–607. 10.1038/s41569-020-0339-2 32080423 PMC7849055

[B53] Tong M. Saito T. Zhai P. Oka S. I. Mizushima W. Nakamura M. (2019). Mitophagy is essential for maintaining cardiac function during high fat diet-induced diabetic cardiomyopathy. Circ. Res. 124 (9), 1360–1371. 10.1161/CIRCRESAHA.118.314607 30786833 PMC6483841

[B54] Wang P. Cui Y. Ren Q. Yan B. Zhao Y. Yu P. (2021). Mitochondrial ferritin attenuates cerebral ischaemia/reperfusion injury by inhibiting ferroptosis. Cell Death Dis. 12 (5), 447. 10.1038/s41419-021-03725-5 33953171 PMC8099895

[B55] Wang P. Cui Y. Liu Y. Li Z. Bai H. Zhao Y. (2022). Mitochondrial ferritin alleviates apoptosis by enhancing mitochondrial bioenergetics and stimulating glucose metabolism in cerebral ischemia reperfusion. Redox Biol. 57, 102475. 10.1016/j.redox.2022.102475 36179435 PMC9526171

[B56] Wang X. Chen X. Zhou W. Men H. Bao T. Sun Y. (2022). Ferroptosis is essential for diabetic cardiomyopathy and is prevented by sulforaphane *via* AMPK/NRF2 pathways. Acta Pharm. Sin. B 12 (2), 708–722. 10.1016/j.apsb.2021.10.005 35256941 PMC8897044

[B57] Wischhof L. Scifo E. Ehninger D. Bano D. (2022). AIFM1 beyond cell death: an overview of this OXPHOS-inducing factor in mitochondrial diseases. EBioMedicine 83, 104231. 10.1016/j.ebiom.2022.104231 35994922 PMC9420475

[B58] Wong N. D. Sattar N. (2023). Cardiovascular risk in diabetes mellitus: epidemiology, assessment and prevention. Nat. Rev. Cardiol. 20 (10), 685–695. 10.1038/s41569-023-00877-z 37193856

[B59] Wu X. Zhou X. Lai S. Liu J. Qi J. (2022). Curcumin activates Nrf2/HO-1 signaling to relieve diabetic cardiomyopathy injury by reducing ROS *in vitro* and *in vivo* . FASEB J. 36 (9), e22505. 10.1096/fj.202200543RRR 35971779

[B60] Wu H. Zhang P. Zhou J. Hu S. Hao J. Zhong Z. (2024). Paeoniflorin confers ferroptosis resistance by regulating the gut microbiota and its metabolites in diabetic cardiomyopathy. Am. J. Physiol. Cell Physiol. 326 (3), C724–C741. 10.1152/ajpcell.00565.2023 38223927

[B61] Xiang Z. Guan H. Zhao X. Xie Q. Xie Z. Cai F. (2024). Dietary gallic acid as an antioxidant: a review of its food industry applications, health benefits, bioavailability, nano-delivery systems, and drug interactions. Food Res. Int. 180, 114068. 10.1016/j.foodres.2024.114068 38395544

[B62] Yan X. Zhang Y.-L. Zhang L. Zou L. X. Chen C. Liu Y. (2019). Gallic acid suppresses cardiac hypertrophic remodeling and heart failure. Mol. Nutr. Food Res. 63 (5), e1800807. 10.1002/mnfr.201800807 30521107

[B63] Yan X. Xie Y. Liu H. Huang M. Yang Z. An D. (2023). Iron accumulation and lipid peroxidation: implication of ferroptosis in diabetic cardiomyopathy. Diabetol. Metab. Syndr. 15 (1), 161. 10.1186/s13098-023-01135-5 37468902 PMC10355091

[B64] Yanatori I. Nishina S. Kishi F. Hino K. (2023). Newly uncovered biochemical and functional aspects of ferritin. FASEB J. 37 (8), e23095. 10.1096/fj.202300918R 37440196

[B65] Yang H. Guan H. Yang M. Liu Z. Takeuchi S. Yanagisawa D. (2015). Upregulation of mitochondrial ferritin by proinflammatory cytokines: implications for a role in Alzheimer's disease. J. Alzheimers Dis. 45 (3), 797–811. 10.3233/JAD-142595 25624418

[B66] Yang C. Zhang Y. Zhang X. Tang P. Zheng T. Ran R. (2023). An injectable, self-healing, and antioxidant collagen- and hyaluronic acid-based hydrogel mediated with gallic acid and dopamine for wound repair. Carbohydr. Polym. 320, 121231. 10.1016/j.carbpol.2023.121231 37659818

[B67] Yoshida H. Goedert M. (2012). Phosphorylation of microtubule-associated protein tau by AMPK-related kinases. J. Neurochem. 120 (1), 165–176. 10.1111/j.1471-4159.2011.07523.x 21985311

[B68] Yuan Q. Sun Y. Yang F. Yan D. Shen M. Jin Z. (2023). CircRNA DICAR as a novel endogenous regulator for diabetic cardiomyopathy and diabetic pyroptosis of cardiomyocytes. Signal Transduct. Target Ther. 8 (1), 99. 10.1038/s41392-022-01306-2 36882410 PMC9992392

[B69] Zhang T. Liu Q. Gao W. Sehgal S. A. Wu H. (2022). The multifaceted regulation of mitophagy by endogenous metabolites. Autophagy 18 (6), 1216–1239. 10.1080/15548627.2021.1975914 34583624 PMC9225590

[B70] Zhang D. Man D. Lu J. Jiang Y. Ding B. Su R. (2023). Mitochondrial TSPO promotes hepatocellular carcinoma progression through ferroptosis inhibition and immune evasion. Adv. Sci. (Weinh) 10 (15), e2206669. 10.1002/advs.202206669 36994647 PMC10214260

[B71] Zhang N. Yu H. Liu T. Zhou Z. Feng B. Wang Y. (2023). Bmal1 downregulation leads to diabetic cardiomyopathy by promoting Bcl2/IP3R-mediated mitochondrial Ca2+ overload. Redox Biol. 64, 102788. 10.1016/j.redox.2023.102788 37356134 PMC10320280

[B72] Zhang L. Luo Y. Lv L. Chen S. Liu G. Zhao T. (2023). TRAP1 inhibits MARCH5-mediated MIC60 degradation to alleviate mitochondrial dysfunction and apoptosis of cardiomyocytes under diabetic conditions. Cell Death Differ. 30 (10), 2336–2350. 10.1038/s41418-023-01218-w 37679468 PMC10589223

[B73] Zhang Y. Zhang J. Sun Z. Wang H. Ning R. Xu L. (2023). MAPK8 and CAPN1 as potential biomarkers of intervertebral disc degeneration overlapping immune infiltration, autophagy, and ceRNA. Front. Immunol. 14, 1188774. 10.3389/fimmu.2023.1188774 37325630 PMC10266224

[B74] Zhao Y. Pan B. Lv X. Chen C. Li K. Wang Y. (2023). Ferroptosis: roles and molecular mechanisms in diabetic cardiomyopathy. Front. Endocrinol. (Lausanne) 14, 1140644. 10.3389/fendo.2023.1140644 37152931 PMC10157477

[B75] Zhong L. Hou X. Tian Y. Fu X. (2025). Exercise and dietary interventions in the management of diabetic cardiomyopathy: mechanisms and implications. Cardiovasc Diabetol. 24 (1), 159. 10.1186/s12933-025-02702-y 40205621 PMC11983742

