# Supplementary figures and images for: Gallic acid attenuates diabetic cardiomyopathy by inhibiting ferroptosis and protecting mitochondria via the TSPO/FTMT pathway
Source: Front Pharmacol. 2025 Dec 3;16:1661144. doi: 10.3389/fphar.2025.1661144 (PMC12708275; doi:10.3389/fphar.2025.1661144)

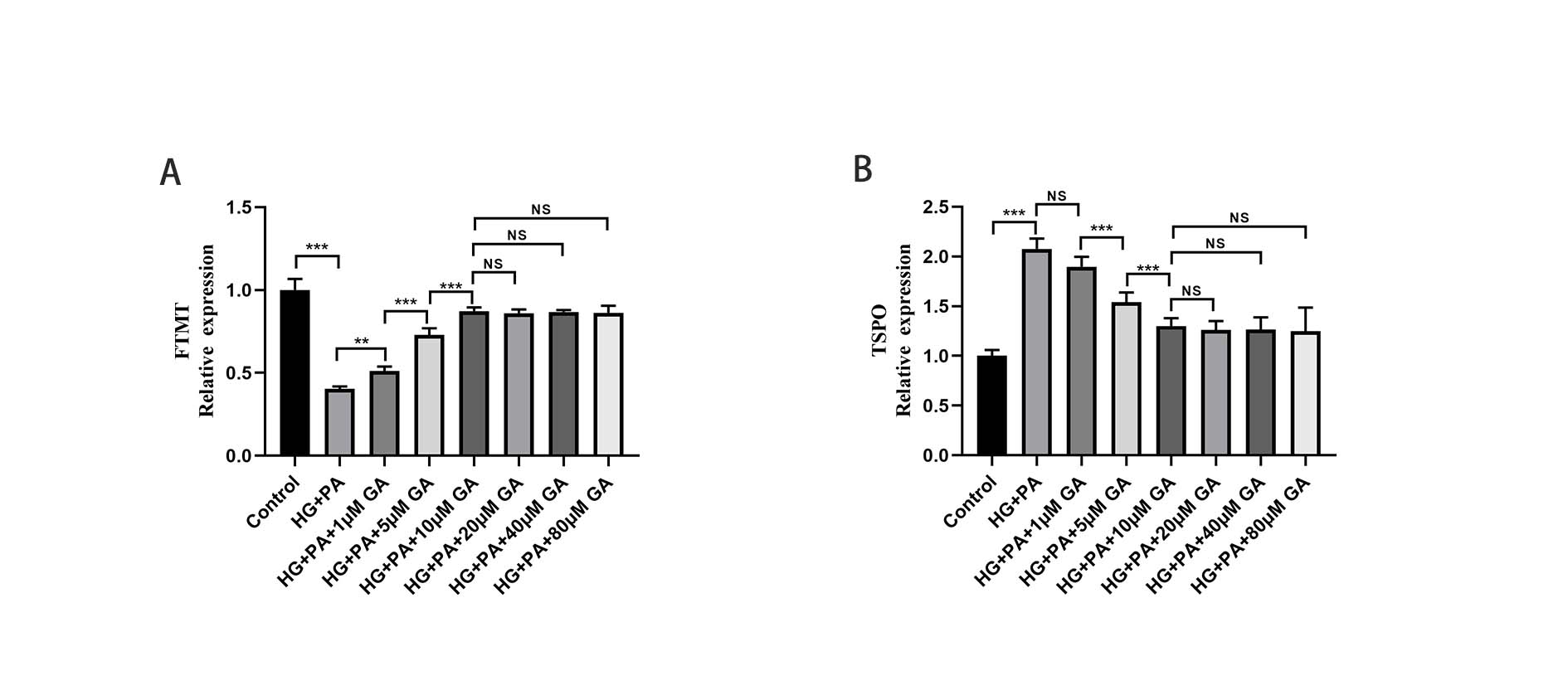

Supplement: Supplementary file 2 [file Image3.jpeg]

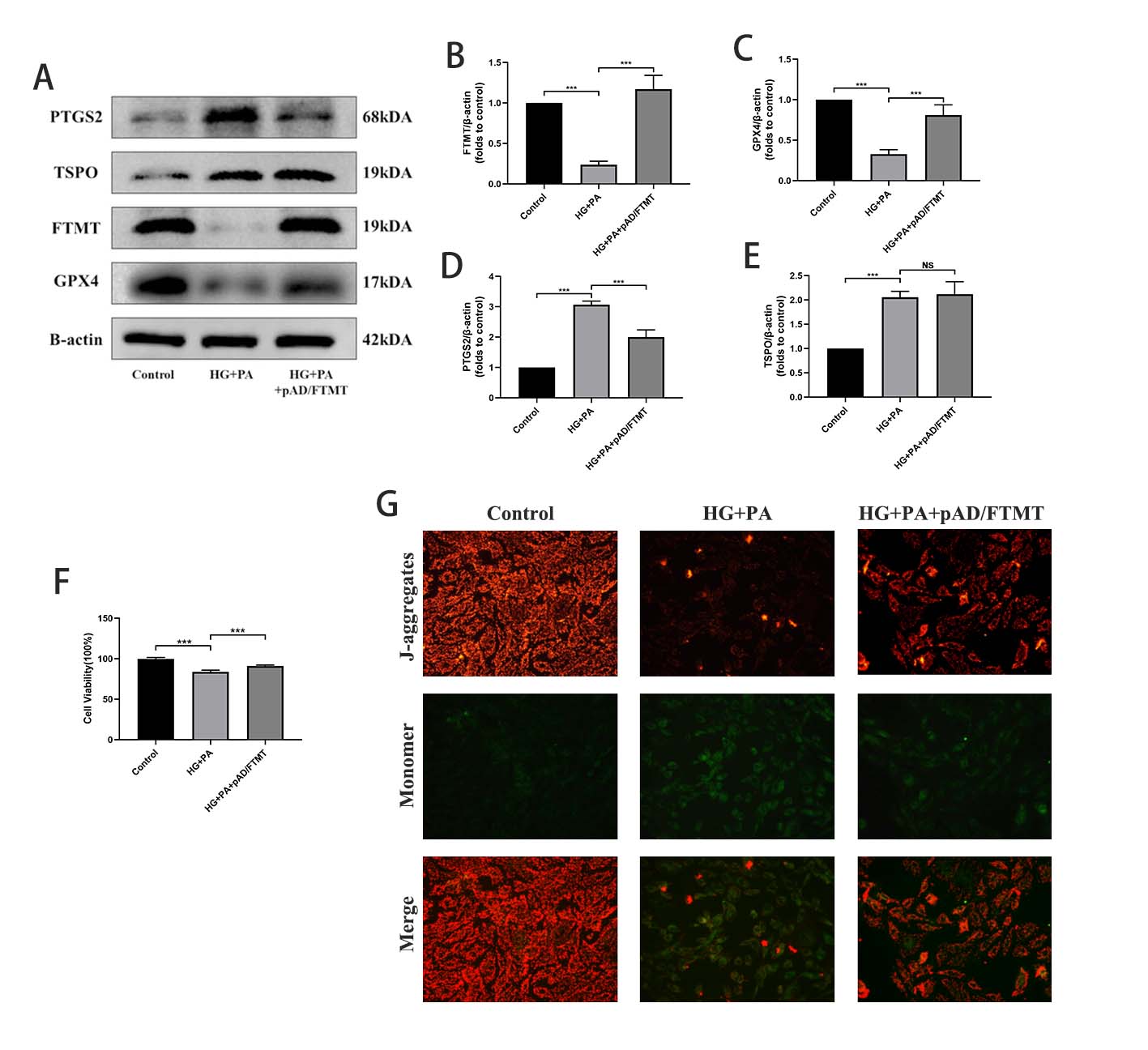

Supplement: Supplementary file 4 [file Image4.jpeg]

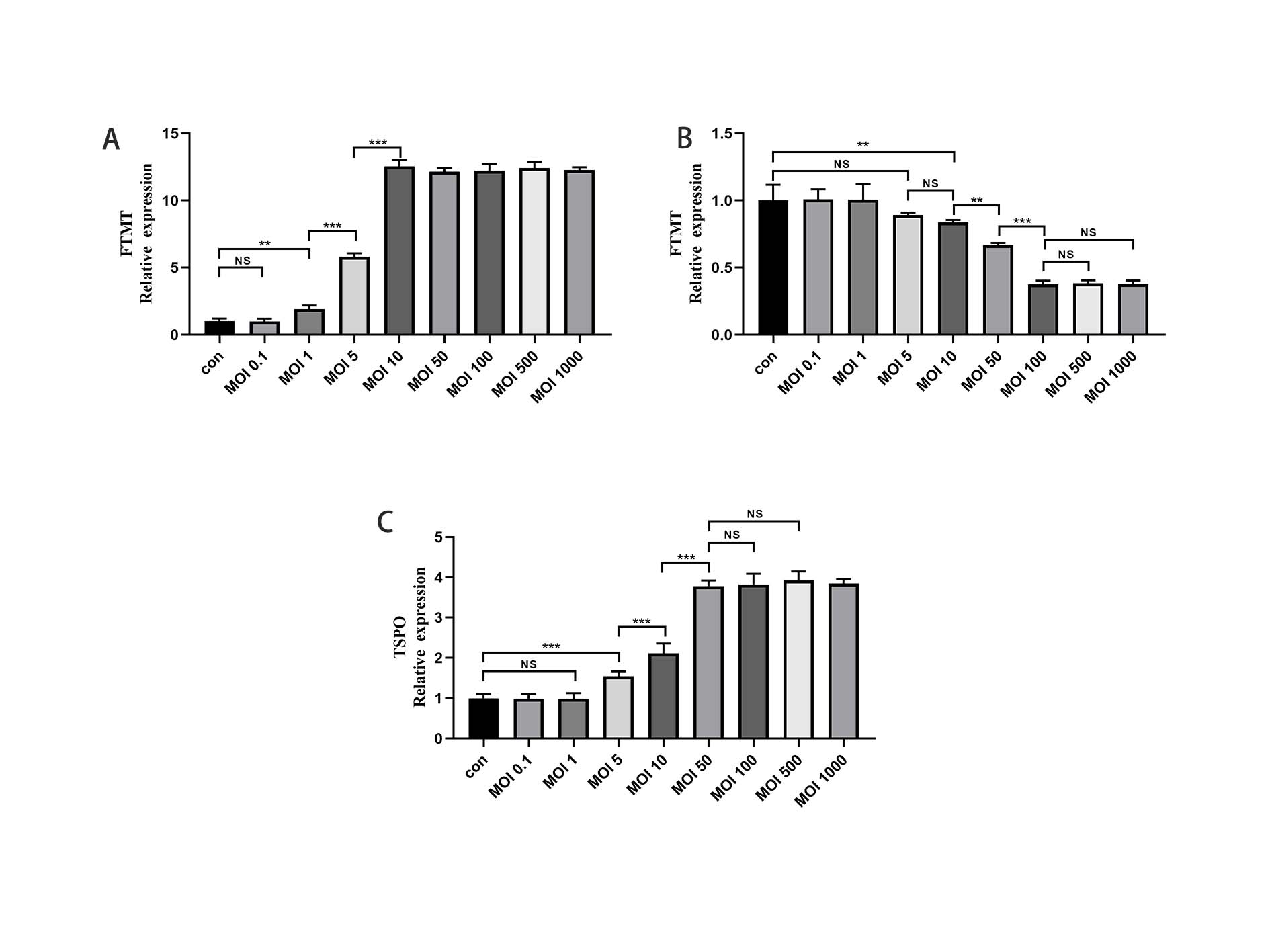

Supplement: Supplementary file 5 [file Image2.jpeg]

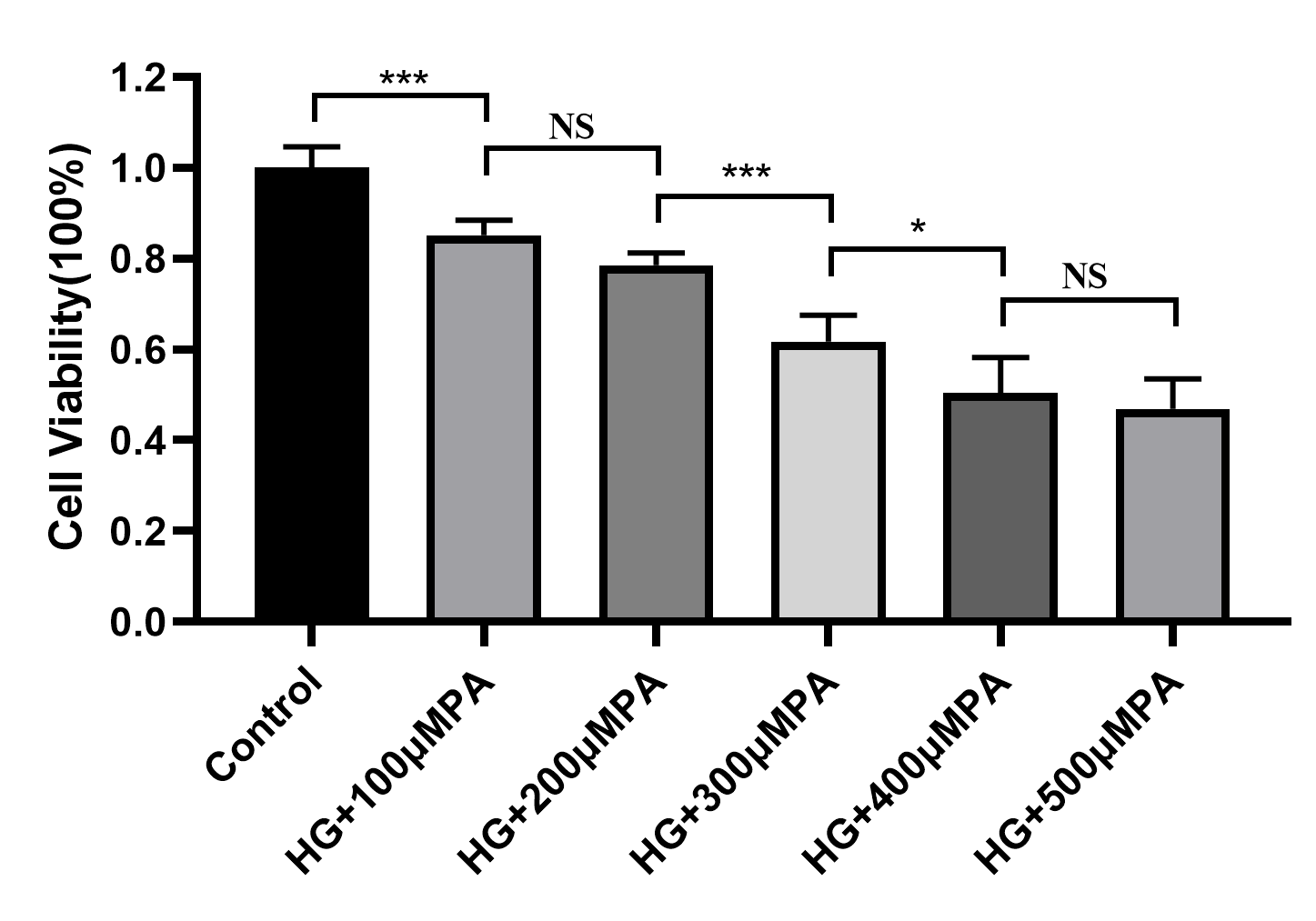

Supplement: Supplementary file 6 [file Image1.tif]

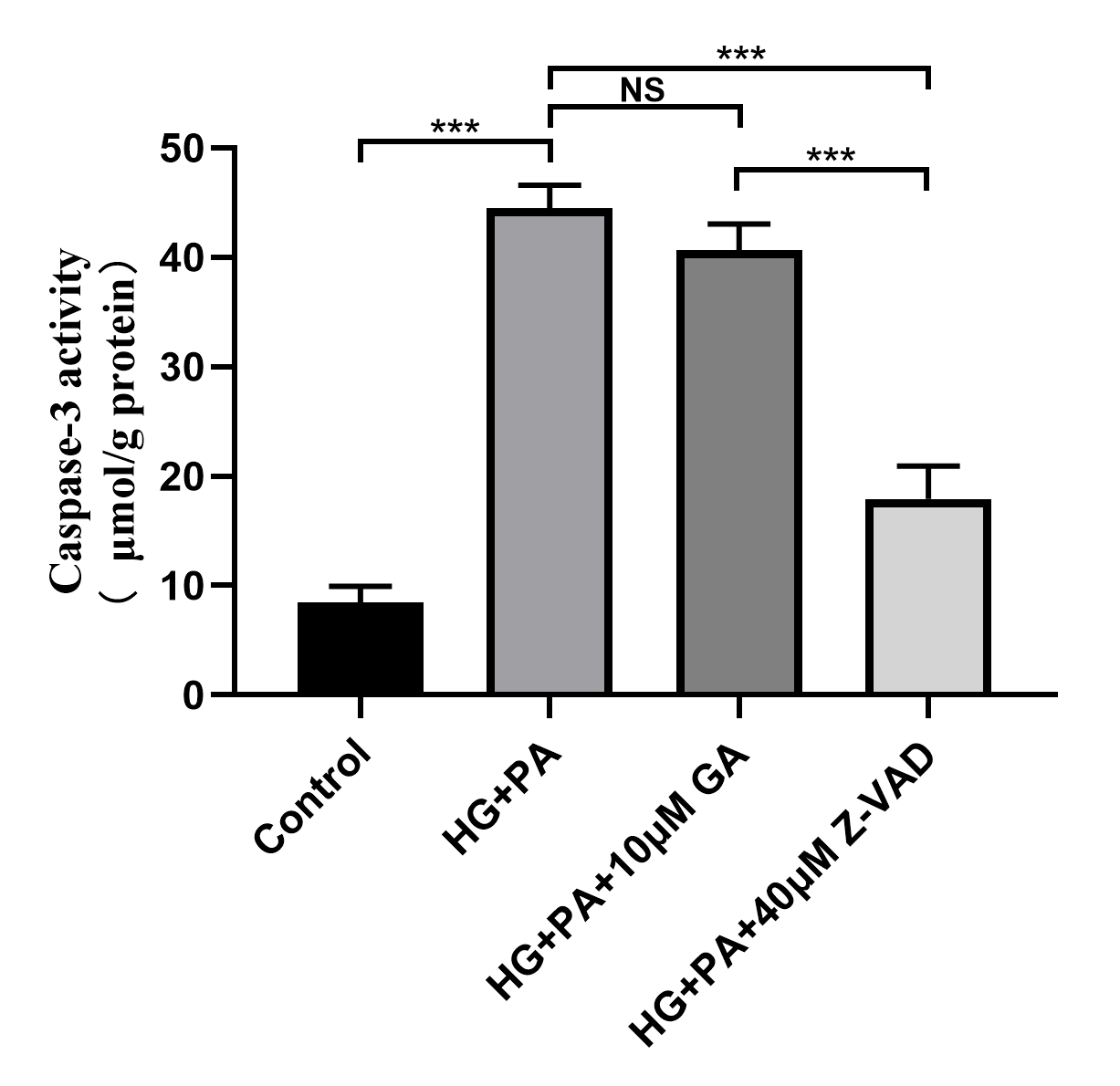

Supplement: Supplementary file 8 [file Image5.tif]

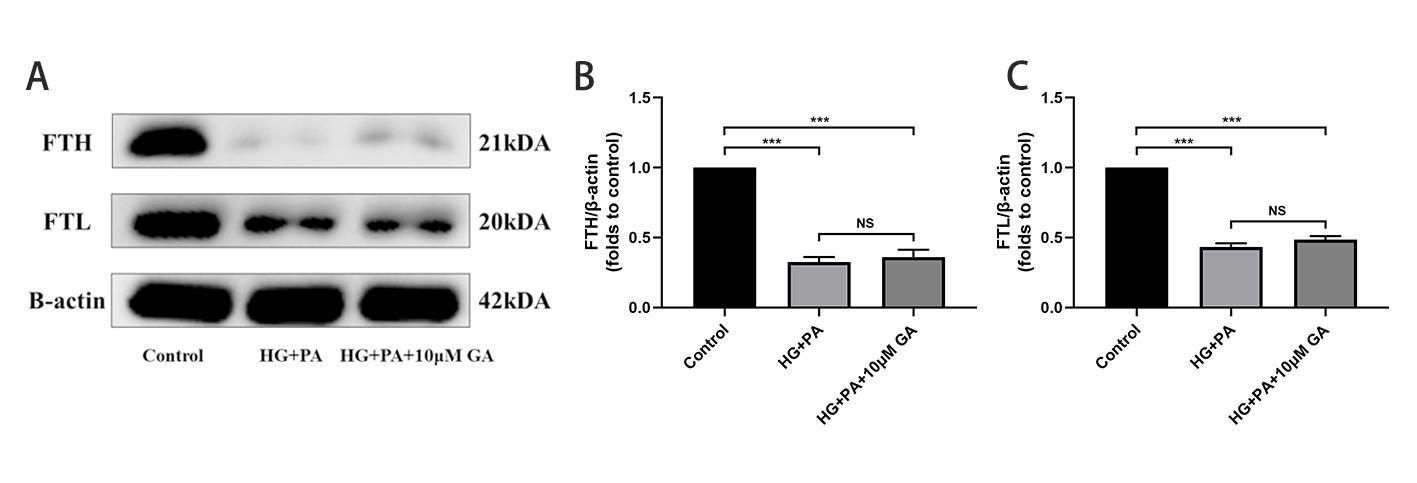

Supplement: Supplementary file 9 [file Image6.jpeg]
