# Supplementary material for: Gallic acid attenuates diabetic cardiomyopathy by inhibiting ferroptosis and protecting mitochondria via the TSPO/FTMT pathway
Source: Front Pharmacol. 2025 Dec 3;16:1661144. doi: 10.3389/fphar.2025.1661144 (PMC12708275; doi:10.3389/fphar.2025.1661144)
Supplement: Supplementary file 3 [file Presentation1.pptx]

## Slide 1
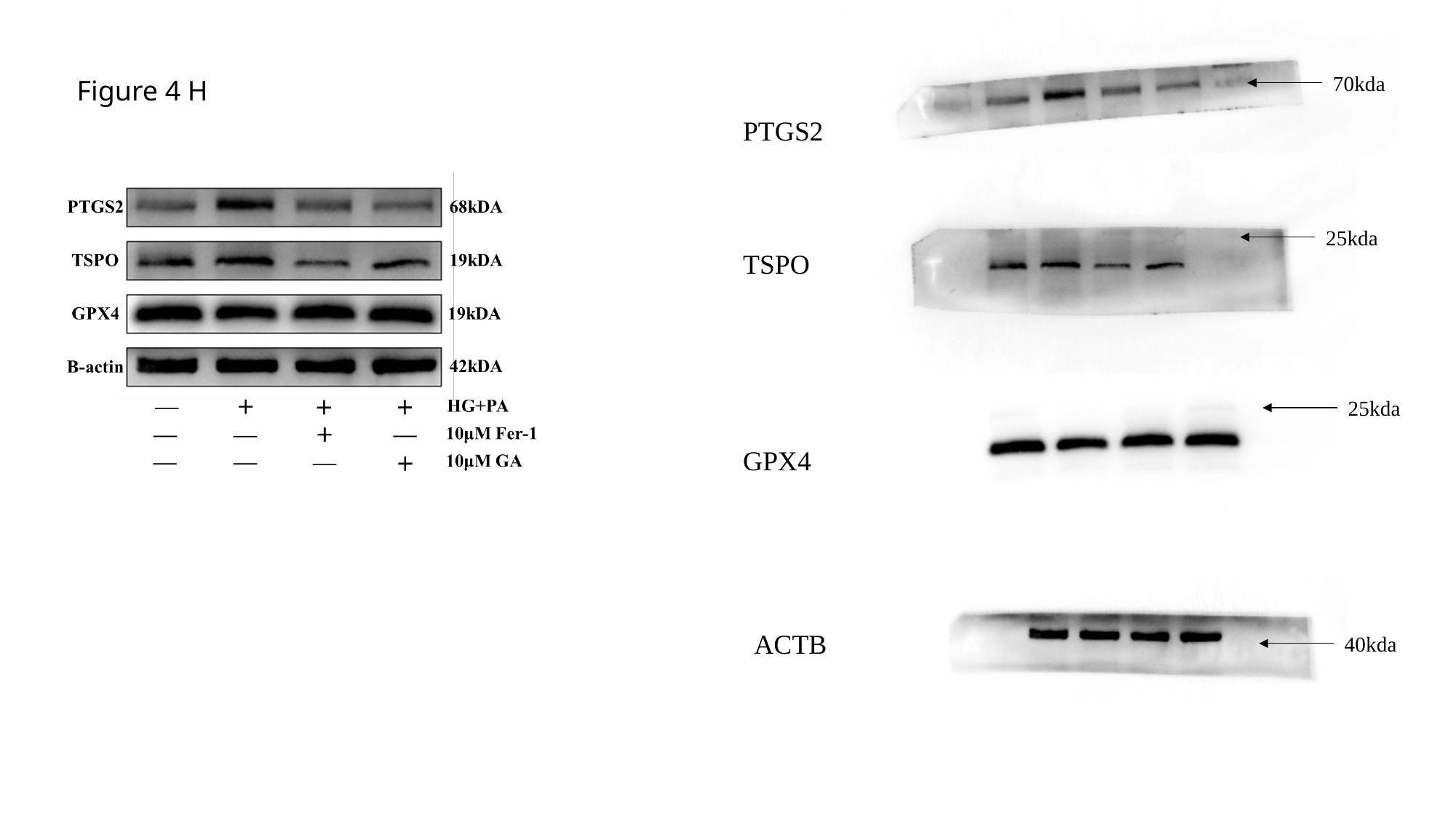

70kda
Figure 4 H
PTGS2
25kda
TSPO
25kda
GPX4
ACTB
40kda

## Slide 2
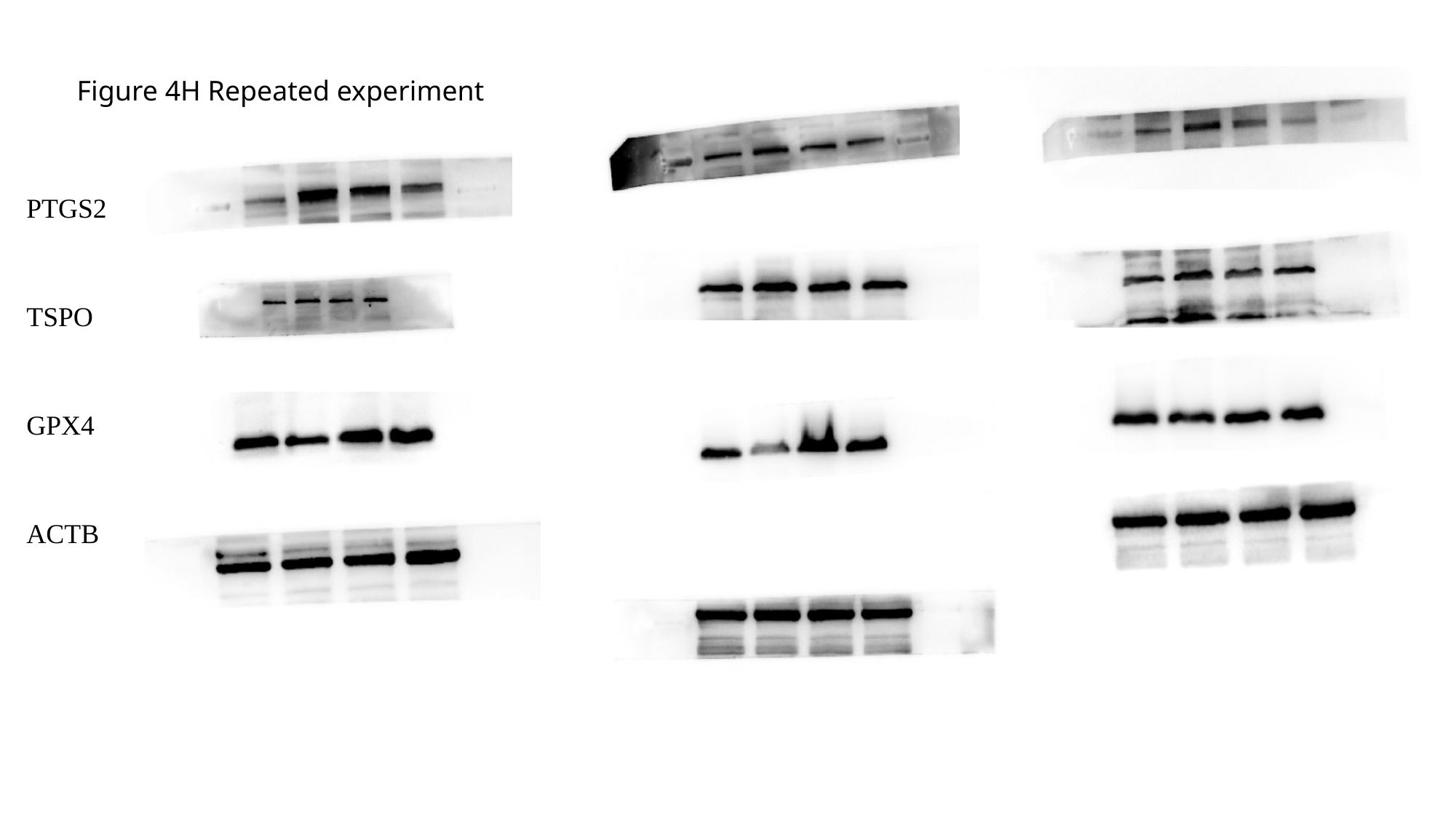

Figure 4H Repeated experiment
PTGS2
TSPO
GPX4
ACTB

## Slide 3
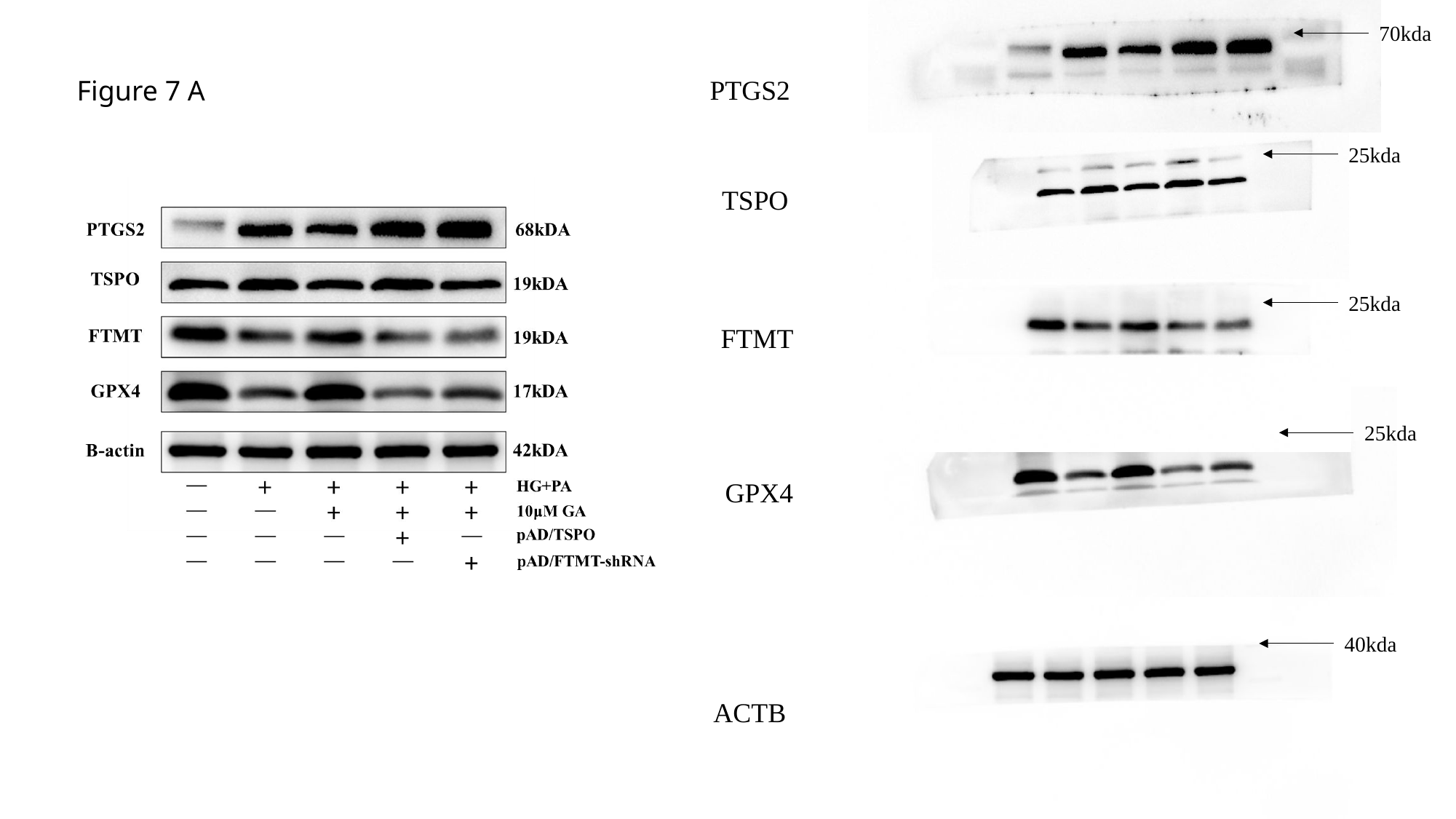

70kda
PTGS2
Figure 7 A
25kda
TSPO
25kda
FTMT
25kda
GPX4
40kda
ACTB

## Slide 4
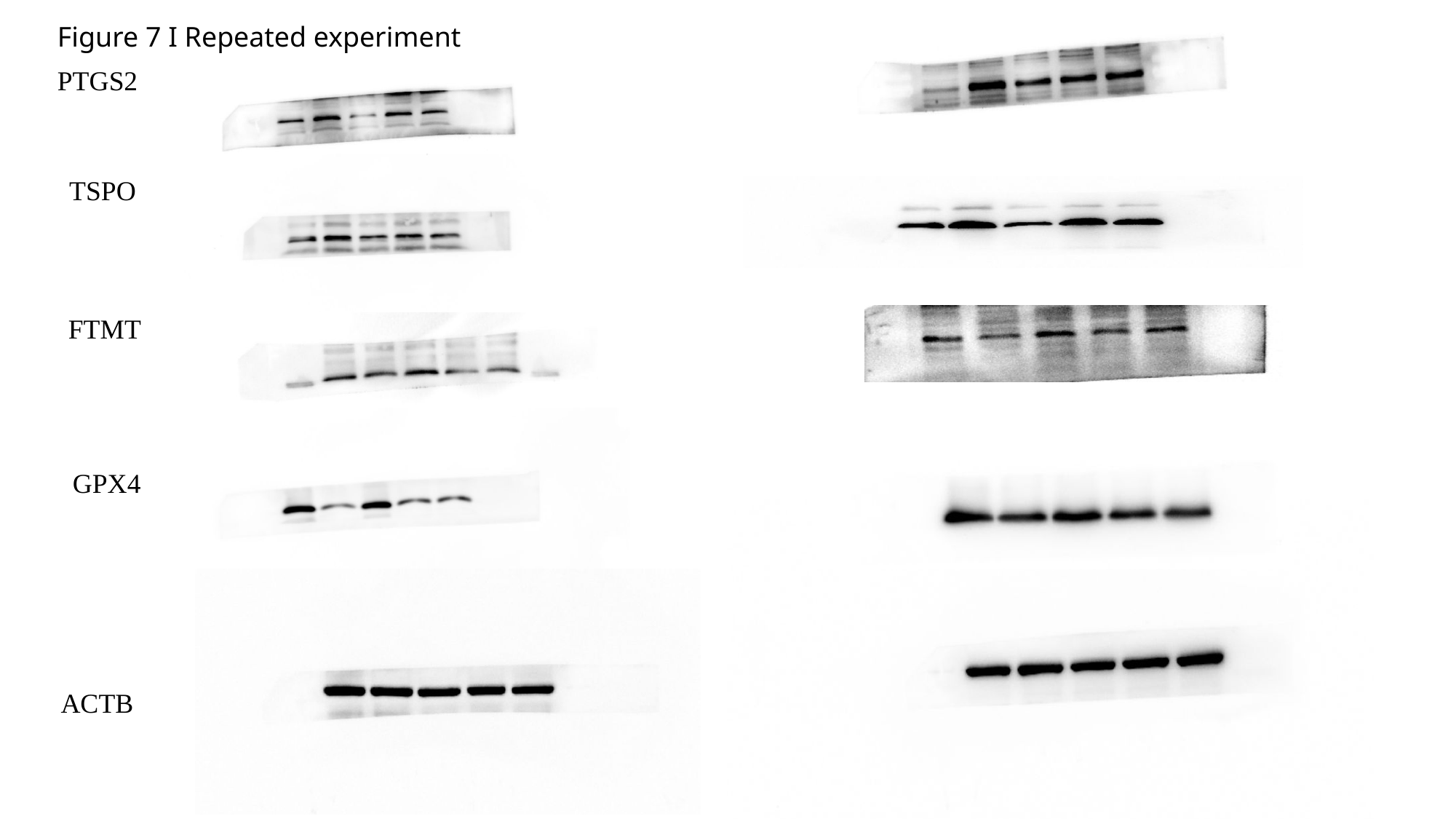

Figure 7 I Repeated experiment
PTGS2
TSPO
FTMT
GPX4
ACTB

## Slide 5
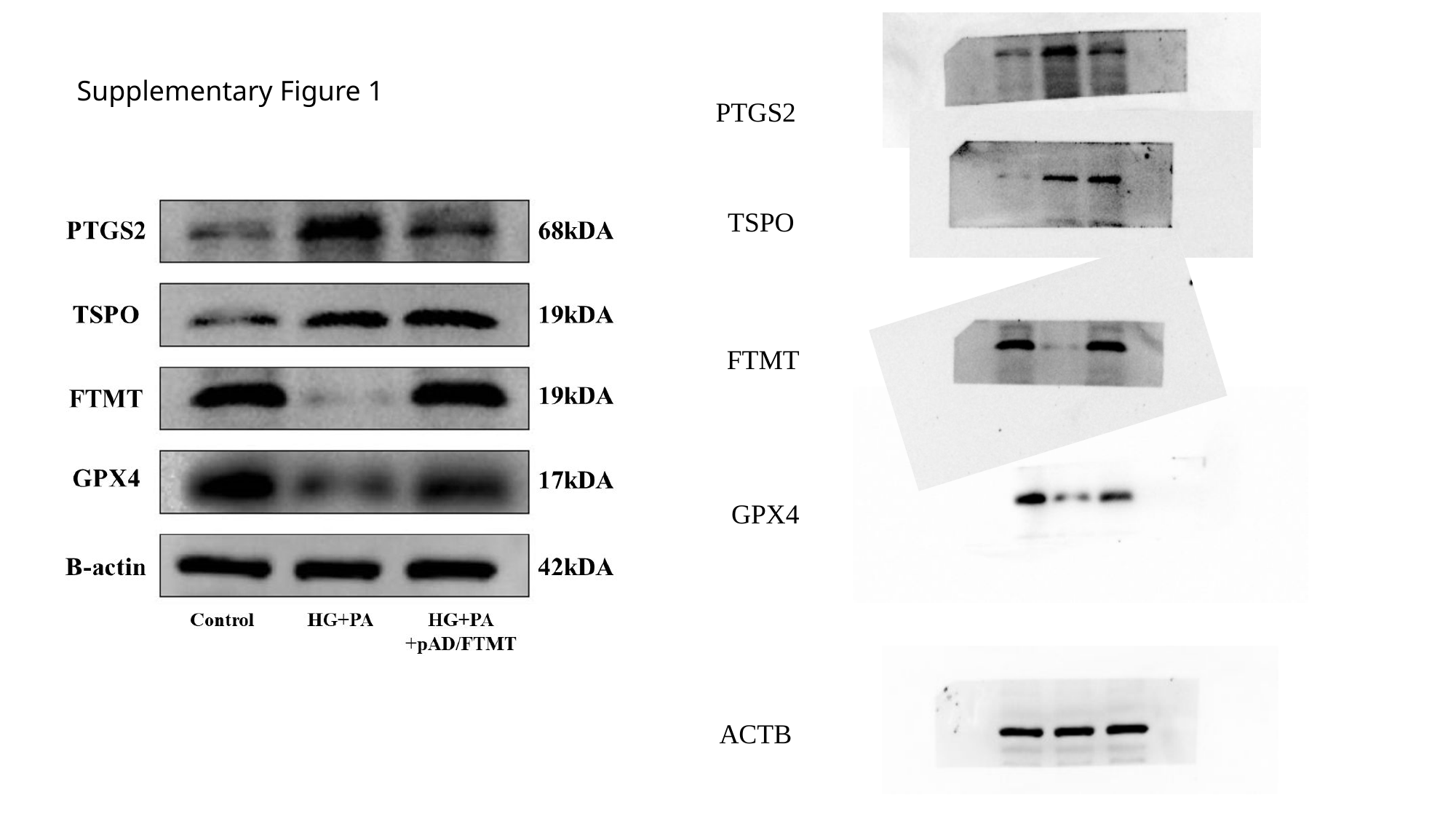

Supplementary Figure 1
PTGS2
TSPO
FTMT
GPX4
ACTB

## Slide 6
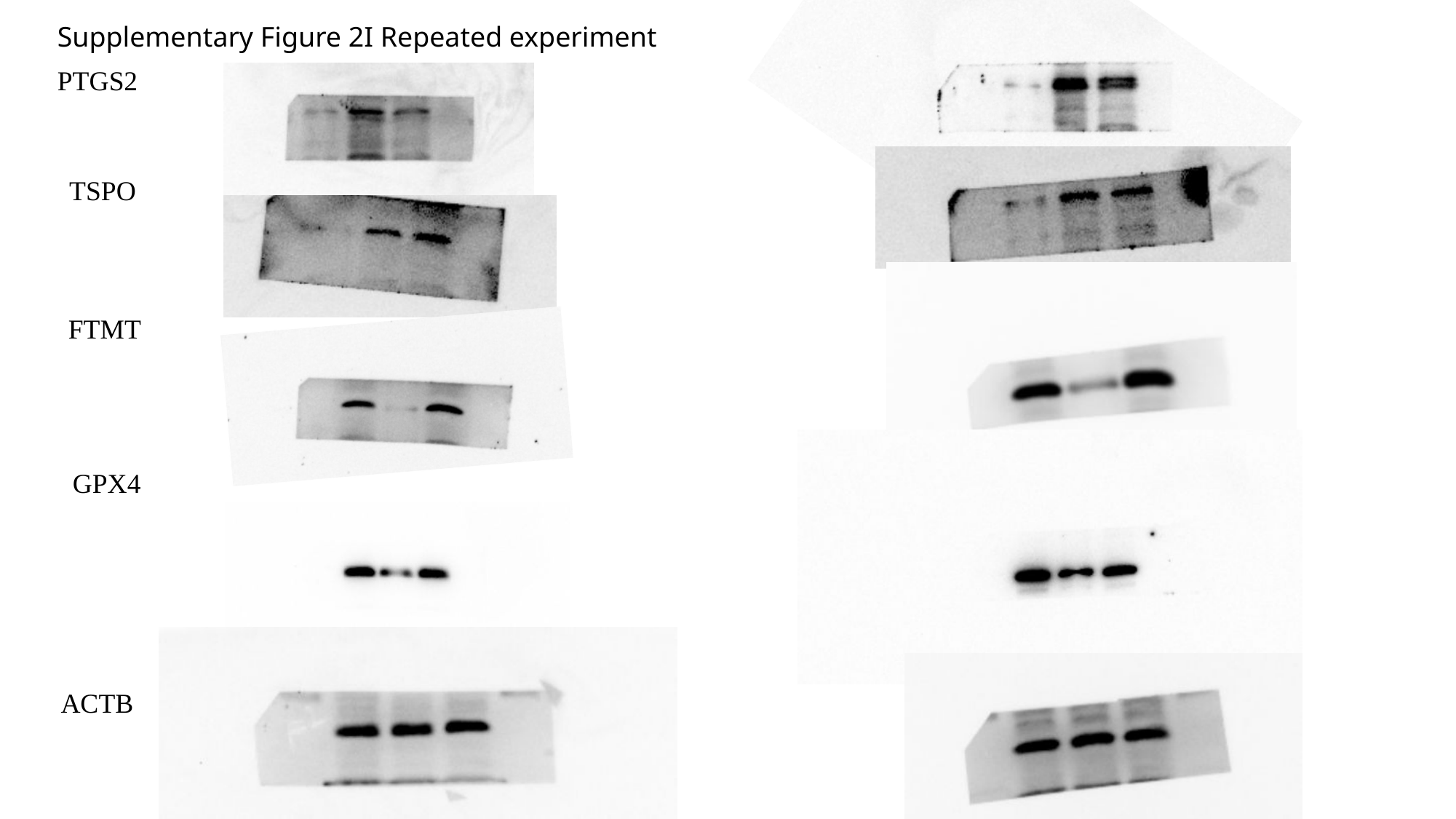

Supplementary Figure 2I Repeated experiment
PTGS2
TSPO
FTMT
GPX4
ACTB
